# Social-ecological network analysis for sustainability sciences: a systematic review and innovative research agenda for the future

**DOI:** 10.1088/1748-9326/ab2619

**Published:** 2019-08-23

**Authors:** J.S. Sayles, M. Mancilla Garcia, M. Hamilton, S.M. Alexander, J.A. Baggio, A.P. Fischer, K. Ingold, G.R. Meredith, J. Pittman

**Affiliations:** 1 ORISE Fellow Appointed with the U.S. Environmental Protection Agency, Office of Research and Development, National Health and Environmental Effects Research Laboratory, Atlantic Ecology Division, Narragansett, RI, USA.; 2 Stockholm Resilience Centre, Stockholm University, Stockholm, Sweden; 3 School of Environment and Natural Resources, The Ohio State University, Columbus, OH, USA; 4 Environmental Change and Governance Group, Faculty of Environment, University of Waterloo, Waterloo, Ontario, Canada; 5 Department of Political Science, University of Central Florida, Orlando, FL, 32816, USA; 6 Sustainable Coastal Systems Cluster, National Center for Integrated Coastal Research, University of Central Florida, Orlando, FL, 32816, USA; 7 School for Environment and Sustainability, University of Michigan, Ann Arbor, MI, USA; 8 Institute of Political Science and Oeschger Centre for Climate Change Research, University of Bern, Switzerland; 9 Department of Environmental Social Sciences, Eawag, Aquatic Research, Dübendorf, Switzerland; 10 Department of Environment and Society, Quinney College of Natural Resources, Utah State University, Logan, UT, USA; 11 School of Planning, University of Waterloo, Waterloo, Ontario, Canada

**Keywords:** Social-ecological networks, human-environment research, social-ecological systems, environmental governance, natural resource management

## Abstract

Social-ecological network (SEN) concepts and tools are increasingly used in human-environment and sustainability sciences. We take stock of this budding research area to further show the strength of SEN analysis for complex human-environment settings, identify future synergies between SEN and wider human-environment research, and provide guidance about when to use different kinds of SEN approaches and models. We characterize SEN research along a spectrum specifying the degree of explicit network representation of system components and dynamics. We then systematically review one end of this spectrum, what we term “fully articulated SEN” studies, which specifically model unique social and ecological units and relationships. Results show more focus on methodological advancement and applied ends. While there has been some development and testing of theories, this remains an area for future work and would help develop SENs as a unique field of research, not just a method. Authors have studied diverse systems, while mainly focused on the problem of social-ecological fit alongside a scattering of other topics. There is strong potential, however, to engage other issues central to human-environment studies. Analyzing the simultaneous effects of multiple social, environmental, and coupled processes, change over time, and linking network structures to outcomes are also areas for future advancement. This review provides a comprehensive assessment of (fully articulated) SEN research, a necessary step that can help scholars develop comparable cases and fill research gaps.

## 1.0 Introduction

Society’s biggest environmental challenges transcend traditional forms of management and require new approaches (DeFries and Nagendra 2017). Environmental issues, such as food, energy, and water sustainability, are often addressed in isolation despite being highly interdependent (Le Blanc 2015). Critical activities, such as watershed restoration, migratory species conservation, or fisheries management, transcend multiple administrative regions, but are all too often dealt with by administrations working in isolation (DeFries and Nagendra 2017). Many of these cross-sectoral, multi-level, and trans-boundary challenges can be addressed by looking at networks of people and the environment (Janssen *et al* 2006, Norberg and Cumming 2008, Bixler *et al* 2016).

A network perspective focuses on relationships. For example, network studies might investigate how one kind of relationship, like trust, explains another relationship, such as interjurisdictional watershed collaborations (Berardo and Scholz 2010, Lubell *et al* 2014). Network studies also focus on how relationship patterns, or structure, affect processes and outcomes. For example, partnership patterns among habitat managers in different municipalities will affect if and how information is exchanged and how projects are coordinated (Bodin and Crona 2009, Dakos *et al* 2015). Structure must, of course, be understood within context. The same network pattern might enhance coordination when parties trust one another, or be co-opted for personal gain when plans are uncertain (McAllister *et al* 2017). In ecological systems, habitat connectivity can enhance the spread of environmental disturbances like fire or invasive species, but also be critical for habitat recovery (Dakos *et al* 2015). Network science (here we include traditions from social, natural, and complex systems sciences) offers a unique conceptualization of the world, complete with theoretical constructs, methods, and tools (Bascompte 2009, Borgatti *et al* 2009, Butts 2009).

Numerous research communities within the wider human-environment^1^ studies are looking to network concepts and methods to advance their work. Examples include, but are not limited to, teleconnections and ecosystem services research (Bohan 2016, Eakin *et al* 2014, Liu *et al* 2015). The network community has also demonstrated how network approaches can advance human-environment studies by addressing issues such as resource exchange (Baggio *et al* 2016), collective action (Lubell *et al* 2014), governing the food-energy-water nexus (Stein and Jaspersen 2018), coordination, and cooperation (Angst *et al* 2018, McAllister *et al* 2017), to name but a few examples. Most network research to date has focused on separate social or ecological networks and their implications for environmental management, such as patterns of interaction among organizations concerned with wildfire risk (Fischer and Jasny 2017), or a network of wildfire-prone habitat patches in which fire spreads (Ager *et al* 2017). Recent advancements, however, have demonstrated that integrated social-ecological networks (SENs), which represent society, the environment, and their interdependencies, can advance our understanding of social-ecological systems (Bodin and Tengö 2012, Bodin 2017).

SENs were first proposed as a way to study social-ecological systems more than a decade ago (Janssen *et al* 2006, Norberg and Cumming 2008, Cumming *et al* 2010). The 2000’s saw little empirical work on SENs however, with Ekstrom and Young (2009) providing one of the first example of how SENs could be used in a concrete case. Despite this slow start, SEN research has now gained its stride with a number of published papers in recent years.

There has been some stocktaking of SEN research with specific topical foci. This work illustrates, for example, how collaboration among users of shared resources leads to successful management, while tests of other theories, such as the benefit of spatial alignment between social collaborations and ecosystem, has mixed results (Bodin 2017). Synthesis work illustrates what social-ecological patterns are likely to facilitate adaptations and transformations (Barnes *et al* 2017). Several commentaries develop ideas to understand ecosystem services outcomes using SENs (Bodin *et al* 2017, Dee *et al* 2017).^2^ While incredibly important within their respective foci, none of these works cover all topics addressed by SEN scholarship, nor do they address higher-order questions about the strengths and limitations of different SEN approaches for environmental problem solving. A comprehensive review about the use of SENs to study various environmental problems is critical given the growing interest in network approaches. Some network methodologies and epistemologies are discussed by Turnbull *et al* (2018) through a fascinating synthesis across disparate scientific disciplines. Turnbull *et al* (2018), however, only briefly mention SENs and do not focus on the science and practice of environmental change, management, and decision making, leaving much ground that still needs to be covered.

**Figure 1.**
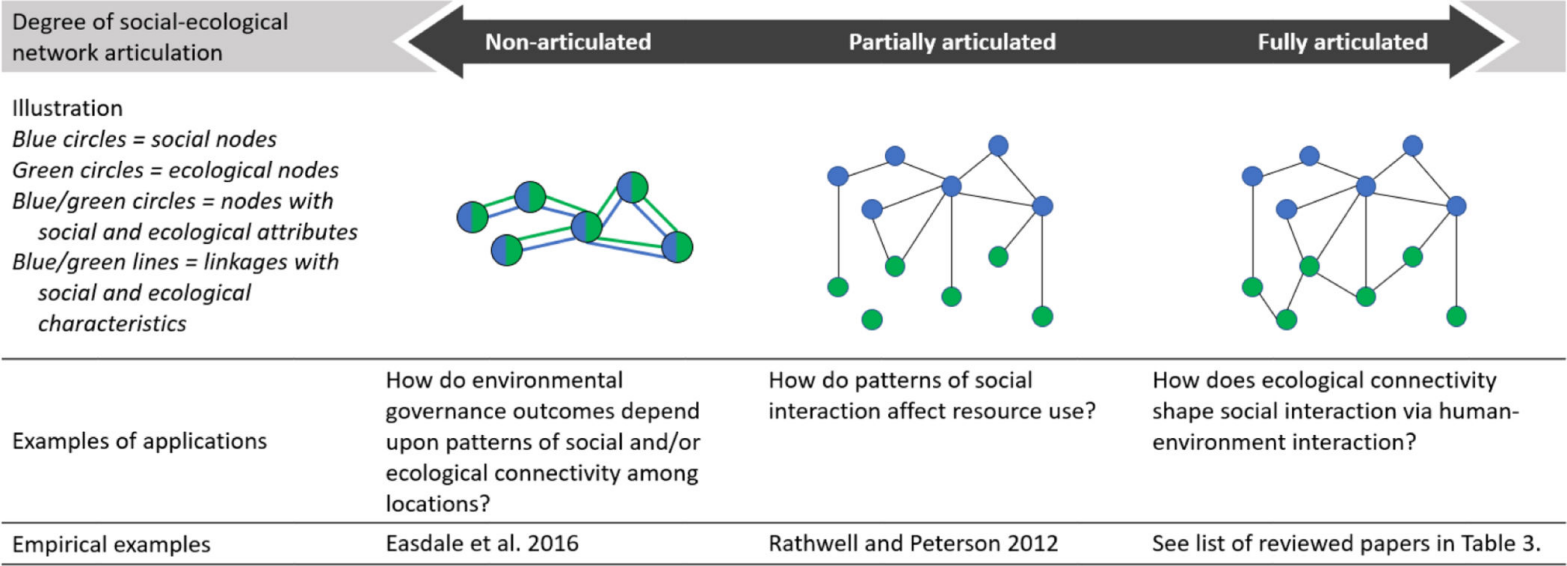
Social-ecological networks (SENs) characterized along a spectrum specifying the degree of explicit network representation of system components and dynamics.

Because SEN scholarship is nascent, this review starts by defining the very field of research to be reviewed. The phrase “social-ecological network” has been applied in a variety of ways to study social-ecological systems (e.g., Rathwell and Peterson 2012, Bodin and Tengö 2012, Baggio *et al* 2016, Easdale *et al* 2016, Sayles and Baggio 2017a). Ambiguous use of this term is undoubtedly confusing. Section 2.0 of this review develops a characterization to situate SEN research along a spectrum specifying the degree of explicit network representation of system components and dynamics (Fig. 1). We then systematically review one end of this spectrum, what we term “fully articulated SEN” studies (section 4.0), those that specifically model unique social and ecological components and relationships. We focus on fully articulated SENs for their potential to uncover fundamental properties of social-ecological systems (Bodin and Tengö 2012, Bodin 2017). We first assess how fully articulated SEN studies have been framed and conducted (section 4.1) including study objectives, theoretical framing, study bounding, method, and the kinds of data used in the study. We then assess how fully articulated SENs themselves are constructed (section 4.2) including the specific social and ecological “things” and relationships in the network and how they are modeled as networks. From this, we discuss the following (section 5.0): What is the current scope of SEN research and what advancements are likely needed? What fully articulated SEN models seem best for different environmental research issues; and what are some methodological and epistemological implications of using the fully articulated SEN approach? And, finally – coming full circle to the original SEN spectrum – when does it make sense to use the SEN approach, fully articulated or otherwise (i.e., when to SEN)?

## 2.0 Characterizing social-ecological networks

SEN research evolved out of several network science traditions from sociology, political science, physics, and complex systems and has adopted certain network terms to describe social-ecological systems. Entities in a network are called nodes and their relationships are called edges.^3^ By definition, SEN research must study social-ecological entities and relationships. This excludes the large bodies of research on social network analysis and ecological network analysis in the context of natural resource management or social-ecological systems (e.g., Bodin and Crona 2009, Ager *et al* 2017, Groce *et al* 2018). These studies look at social or ecological processes, respectively, as networks, and contextualize them within environmental management. For example, Ager *et al* (2017) model networks of wildfire transmission (ecological connectivity) among forested areas in a multi-jurisdictional landscape in Oregon, USA. Because interjurisdictional management and how jurisdictions interact with fire are not included in the network, it is not a SEN.

Even if nodes represent social and ecological entities, the study still may not be a SEN. A SEN must address both social and ecological phenomena, and their interaction, in a meaningful way. For example, Zhang *et al’s* (2016) study of nitrogen flows in urban systems analyzes a network comprised of social (e.g., household) and ecological (e.g., forest) nodes. This network only includes nitrogen flows (an ecological process), and therefore is akin to an ecological network model that includes households as an ecosystem component. Social processes, concepts, or theories are not considered in the study in any meaningful way, nor do they underpin network conceptualization and therefore the study does not constitute a SEN.

### 2.1. A spectrum of social-ecological networks

Among studies that do account for connectivity among social and ecological entities, there is considerable diversity in how SENs are conceptualized. We view this diversity as a spectrum, from non-articulated to fully articulated SENs, along which SEN approaches can be positioned according to how much they articulate social and ecological components and relationships (Fig. 1). Non-articulated SENs study social-ecological systems as networks, but do not distinguish between social and ecological nodes as discrete entities. The aggregated flow of herders and animals (edges) moving between seasonal habitat areas (nodes) is an example (Easdale *et al* 2016). While certainly a network *of* a social-ecological system, this conceptualization does not reveal specific social and ecological dynamics and those co-produced from social-ecological interactions.

Partially articulated SENs start to disentangle social-ecological components, but do not include all types of relationships among these components (Fig. 1). For example, Rathwell and Peterson (2012) analyze water management outcomes by measuring management collaborations (social edges) and link these to specific places (social to ecological edges), but do not account for relationships among ecosystem components, such as hydrologic connections (ecological edges), which could be used to account for up and downstream power asymmetries among collaborators (Lebel *et al* 2005, Sayles 2018, Herzog and Ingold 2019). This illustrative example omits ecological relations, but a network omitting social relations would also be partially articulated.

Disentangling social, ecological, and social-ecological network relationships results in a fully articulated SEN. An example might be a network of fishers and how they communicate (social interactions) connected to a network of harvested species (social-ecological interactions) and their food-web (ecological interactions) (Bodin 2017). This fully articulated SEN explicitly disentangles social and ecological structures and processes to study interactions (Bodin and Tengö 2012) (Fig. 1).

Fully articulated SENs open up numerous conceptual and analytical possibilities to develop and test theories and measure social-ecological patterns, because all system relationships and dynamics can be considered. For this reason, we restrict our systematic component of the review to studies of fully articulated SENs (Fig. 1); however, we emphasize that different approaches for conceptualizing SENs (e.g., as non-, partially, or fully articulated) depend entirely upon the research questions at hand. We reflect further on working with SENs of different degrees of articulation in section 5.

### 2.2. Different network models

**Figure 2.**
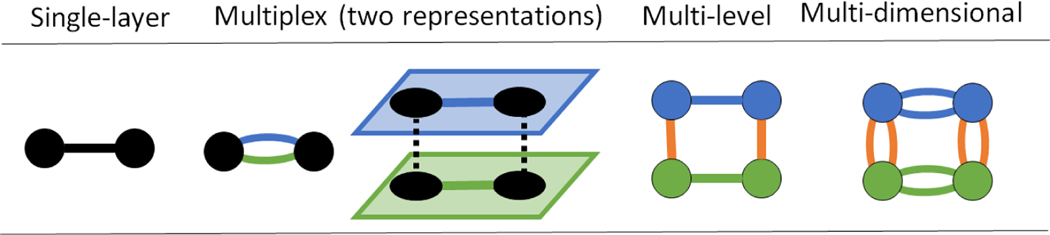
Different kinds of networks. Circles are nodes. Lines are edges. Black nodes/edges are social or ecological, defined by attribute values. Blue, green, and orange are social, ecological, and social-ecological, respectively. Multiplex networks are depicted in two ways: first, where nodes have multiple edges; second, where edges have been extrapolated to different social and ecological layers. The same nodes are present in both layers and their alignment (dashed line) depicts the social-to-ecological relationship. All network types are described in the main text.

Fully articulated SENs can be conceptualized and operationalized in several ways, modeling approaches that we consider in our characterization of the literature (Fig. 2). Each approach contains assumptions about how nodes and edges can be related. The basic model is a *single-layer* network. It is the kind with which most people are familiar, such as a stakeholder network or a food web. Two nodes in a single-layer model may only share one relationship; however, social and ecological nodes and edges can be differentiated graphicly using attribute values (Cumming *et al* 2010). These attributes are lost, however, in single-layer network mathematics, which treat all nodes and edges the same.

Most real-world phenomena are much more nuanced than a single relationship. Accordingly, network scientists have strived to develop models that are better able to represent reality (Fig. 2). *Multiplex* networks allow nodes to be connected through multiple types of relationships, such as information sharing, cultural similarity, and monetary exchange among fishers. Often each of these relationship-types is represented as a unique layer, each containing the same set of nodes, though some may be isolates, having no edges in a given layer (Boccaletti *et al* 2014, Kivelä *et al* 2014). For example, a fully articulated multiplex SEN might represent specific management areas as nodes. Social and ecological relationships among these areas, such as management collaboration and animal movement, would then be represented as unique social and ecological layers. The alignment of these node layers constitutes the social-ecological edges that represent relationships such as management responsibility or resource harvesting.^4^ *Multi-level* networks depict the SEN in a slightly different way by allowing two or more kinds of nodes. Different kinds of nodes can be thought of as network layers, but unlike multiplex networks, the number of nodes can be different in each layer of the multi-level network. Multi-level networks allow different relationships between each type of node, but only one relationship is allowed between any two nodes (Lomi *et al* 2015). A multi-level SEN might represent, for example, social relations among any number of resource users, social-ecological edges connecting them to specific habitat patches that they manage, and edges among those habitat patches depicting ecological connections (Bodin and Tengö 2012). Lastly, *multi-dimensional* networks allow for multiple relationships among multiple kinds of nodes, essentially combining multiplex and multi-level concepts (Shumate and Contractor 2013).^5^

These four types of networks provide a general typology for constructing SENs. They may be mathematically defined and analyzed in many different ways (for example, see note 3, and the review by Kivelä *et al* (2014)). Other types of networks and alternative terms have been defined in the literature, though most, in essence, are variations of those depicted here (Kivelä *et al* 2014). Forcing a consensus on terminology is impossible and most likely unproductive. It is important to understand that alternatives exist, often aligning with specific academic disciplines. With this malleability in mind, our SEN network typology is a good foundation to discuss the implications of translating the world into network models.

Finally, we classify network models as either *landscape* or *systems* approaches. *Landscape approaches* are geographically and spatially explicit, such as those focusing on coordinated management of wetland habitat patches (Bergsten *et al* 2014), watershed restoration (Sayles and Baggio 2017a), or forest fires (Hamilton *et al* 2019); however, SEN approaches (of any articulation) do not need to be rooted in geographic space. The only requirement of a network model is that entities in the system must be represented as nodes and their relationships as edges. Many SENs represent abstract or theoretical social and ecological entities and relationships. For example, Ekstrom and Young (2009) depict a theoretical estuary ecosystem coupled with theoretical human stressors. While based on understandings of real-world estuaries, the network is an abstraction and a-spatial, and what we call a *systems approach*.

## 3.0 Methods

### 3.1 Article Selection

To review fully articulated SEN studies, we performed a topic search in ISI Web of Knowledge (WOK) using search terms that represent a variety of ways for talking about coupled human-environment or social-ecological systems. Joined by Boolean “OR”, we used the following search terms: social-ecological AND network, socio-environmental AND network, socio-ecological AND network, human-environment AND network, “coupled human and natural systems” AND network, CHANS^6^ AND network. This approach ensured a comprehensive inventory of relevant papers independent of the phrase “social-ecological network.” The search, which was last updated on 17 December 2018, returned 1,232 papers (details in figure S1).

The lead author manually screened all papers to remove those that did not specifically address network science and only, for example, talked about the importance of networks or networking in social-ecological systems. Papers were screened using the abstract or main text as necessary; 338 were retained as candidate papers for the review. From this, the team reviewed and discussed an initial subset of purposely selected papers to pilot and refine the inclusion criteria and coding protocol.

Papers were included in the final review if they met the following criteria: 1) They were empirical or substantive studies based on primary field or desk research, case-study synthesis, or computational modeling. Review and opinion papers were not included. 2) Papers needed to undertake the “fully articulated” approach, i.e., have a clear set of social and ecological nodes and contain edges within and between these social and ecological sub-components. These subcomponents did not need to be specifically identified by paper authors, but needed to be identifiable to the coders based on their interpretation. 3) Papers needed to include networks as a system driver or outcome. An agent-based model, for example, that simply included a network component in the model background in order to simulate a more realistic universe, but did not seek to understand how the network shapes environmental outcomes, or how human-environment problems shape the network, would not have been included. For this reason, papers using Bayesian belief networks, or concept mapping, tended to be omitted despite using networks. The 338 papers were reviewed and classified for inclusion in the review by two independent coders, who made a final consensus-based decision.

We complemented our topic search with our own knowledge of the literature and included any articles that were not returned in the WOK search, such as new articles that had not been indexed. As a young and growing body of scholarship, including these most recent papers is essential to understand SEN research’s trajectory. In addition, we reviewed the reference list of all included papers to check whether other papers should be included. No additional papers were identified through this procedure. We acknowledge that there may be papers that were neither included in the WOK search, the reference lists of included papers, or through our own knowledge, but through this triangulation of search approaches we believe to have compiled most, if not all, fully articulated SEN studies in peer-reviewed journals. While omissions are possible, our review illustrates the diversity of uses of the fully articulated SEN approach and the rich sets of possibilities it offers.

### 3.2. Coding and analysis

To understand our first line of inquiry, how fully articulated SEN studies have been framed and conducted, we coded the papers’ objectives, theoretical framing, how the study was bound, the kind of evidence, and methods used. These variables (Table 1, details in Supplemental Information (SI)) directly indicate the objectives of SEN research and how researchers have engaged human-environment topics. Our second line of inquiry, how fully articulated SENs are constructed, focused on the kinds of nodes and edges included in the networks and how the networks were conceptualized according to section 2.2. Categorizing nodes and edges illustrates how researchers conceptualize units of analysis within a given social-ecological system (e.g., individuals vs. organizations vs. institutions) and how they represent social-ecological systems, as SENs (see the discussion in section 2.1). Based on our knowledge of SENs and human-environment research, we developed a set of deductive codes to categorize nodes and edges and allowed for additional write-in responses (Table 2, details in SI).

All SEN papers included in the review were coded by two independent coders. The three first authors then reviewed and resolved any discrepancies among coders through a consensus decision and ensured the codes were applied consistently. Some codes refer to the overall paper (e.g., how it was framed), while others addressed the SEN (e.g., how nodes and edges were defined). Though infrequent, a paper could describe more than one SEN, for example in a comparative case analysis. Therefore, the sample size varies among reported results depending on if they describe the paper or the SEN(s) described in the paper.

### 3.3. Citation network analysis

We also constructed and visualized a citation network to determine if SEN papers were closely linked to each other to provide a broad picture of SEN research. We did not expect all papers to be linked through direct citations, but expected papers to draw from common theoretical works. We therefore visualized SEN papers and their common citations and identified if common citations were key works of theory or simply methodological. See the SI for details.

## 4.0 Results

**Figure 3.**
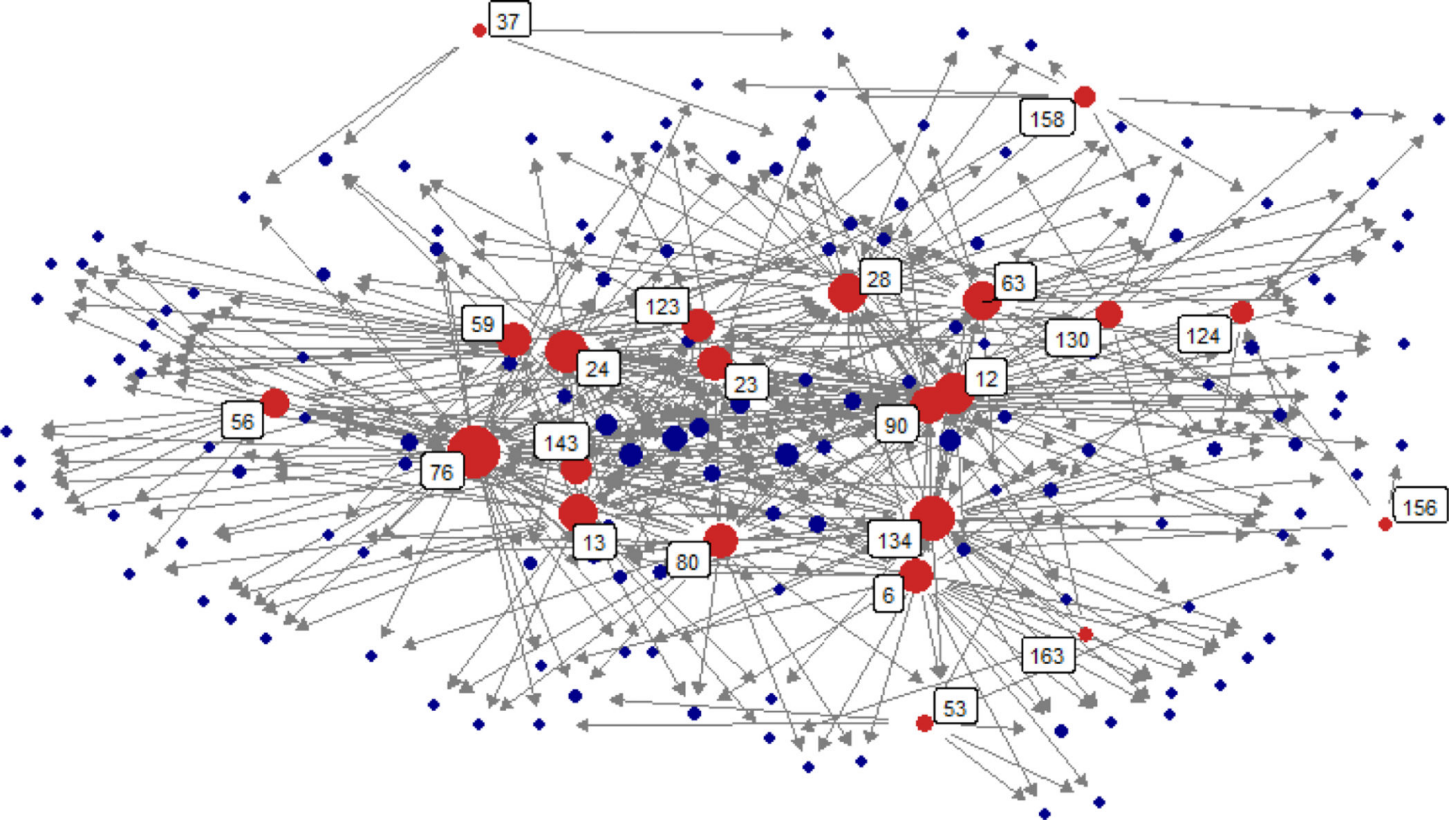
Network diagram showing how the 22 SEN papers (red) are linked directly or through common citations (blue, n = 141; total network = 163 nodes). For clarity, we removed all references that were only cited by a single SEN paper and thus, not uniting the network. As expected, the SEN papers form a cohesive network within one degree of separation. Empirical studies and key works of theory unite the network (Table S1, Fig S6). No two SEN papers were linked solely through methods books or papers revealing a field unified around complex systems and issues like common pool resource governance. Node size represents the total number of cited references (for 22 SEN papers only) plus citations (for all papers) in the network. The 22 SEN papers are labeled using network ID values, which are explained in Table 3. For more details see the SI.

From the original pool of 1,232 papers, we identified 22 fully articulated SEN papers (Table 3) that draw from common theoretical foundations (e.g., Cumming *et al* 2006, Folke *et al* 2005, 2007, Galaz *et al* 2008, Janssen *et al* 2006, Ostrom 1990, Urban and Keitt 2001, Young 2002; Fig. 3, details in Fig S7, Tabel S1). Most papers were published since 2014.

While 22 papers represent a small scholarly corpus, they nevertheless comprise a large enough body of work to distill emerging patterns and learn how authors approach fully articulated SENs. We must be cautious, however, not to overreach when drawing conclusions. The following sections (4.1. and 4.2.) must be interpreted as highlighting diversity among fully articulated SEN research, its foundation, and its potential for growth.

### 4.1. Study framing and implementation

**Figure 4.**
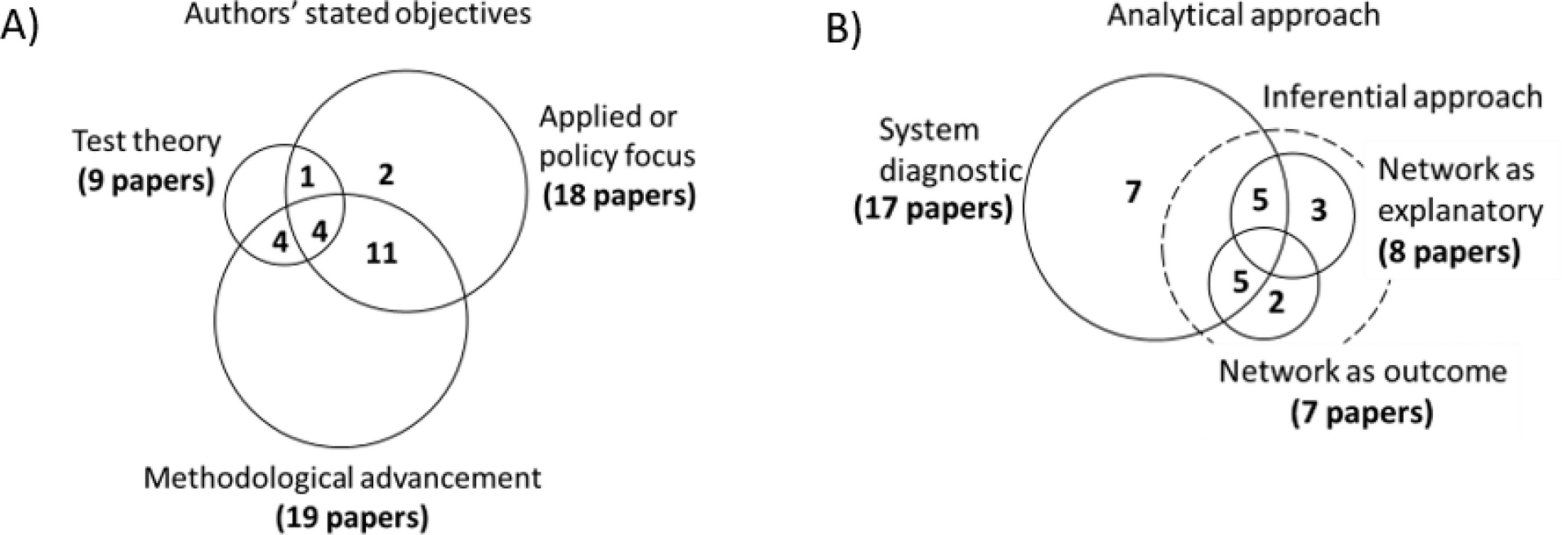
Venn Diagrams of A) author’s stated objectives for the paper and B) analytical approach used. N = 22. Number of papers by category are labeled. Circles are proportional to the total number of papers.

Perhaps indicative of a budding research field, the majority of the 22 fully articulated SEN papers (86.4%) had clearly stated methodological aims. No paper, however, solely hung its hat on methods. Theory testing or applied ends were always motivators, though the latter was much more frequent (Fig. 4a).

Fully articulated SEN papers were split between taking a diagnostic or inferential approach (n = 17 and 15), with ten papers combining the two (Fig. 4b). Inferential approaches were split between those seeking to explain what shapes network structure (network as outcome, n = 7) and how network structure explains social or ecological outcomes (network as explanatory, n = 8). At face value, the relatively high ratio of papers linking network structure to outcomes is promising as strong empirical evidence is scarce in the wider literature on network science for natural resource management and sustainability (Barnes *et al* 2016, Groce *et al* 2018). On closer investigation, however, only three papers clearly link social-ecological structure with case study outcomes and among the three papers, only two unique case studies are presented (Bodin and Tengö 2012, Bodin *et al* 2014, 2016). Three papers are based on model simulations (Chopra and Khanna 2014, Dragicevic and Shogren 2017, Baggio and Hillis 2018); and two papers (Sayles and Baggio 2017a, Yletyinen *et al* 2018) correlate social-ecological structure with indirect proxies of system performance: study participants’ perceptions of management activities and inferred adaptive capacity and vulnerability of resource harvesters, respectively. While efforts have clearly been made, strong evidence linking network structure to outcomes is still lacking.

**Figure 5.**
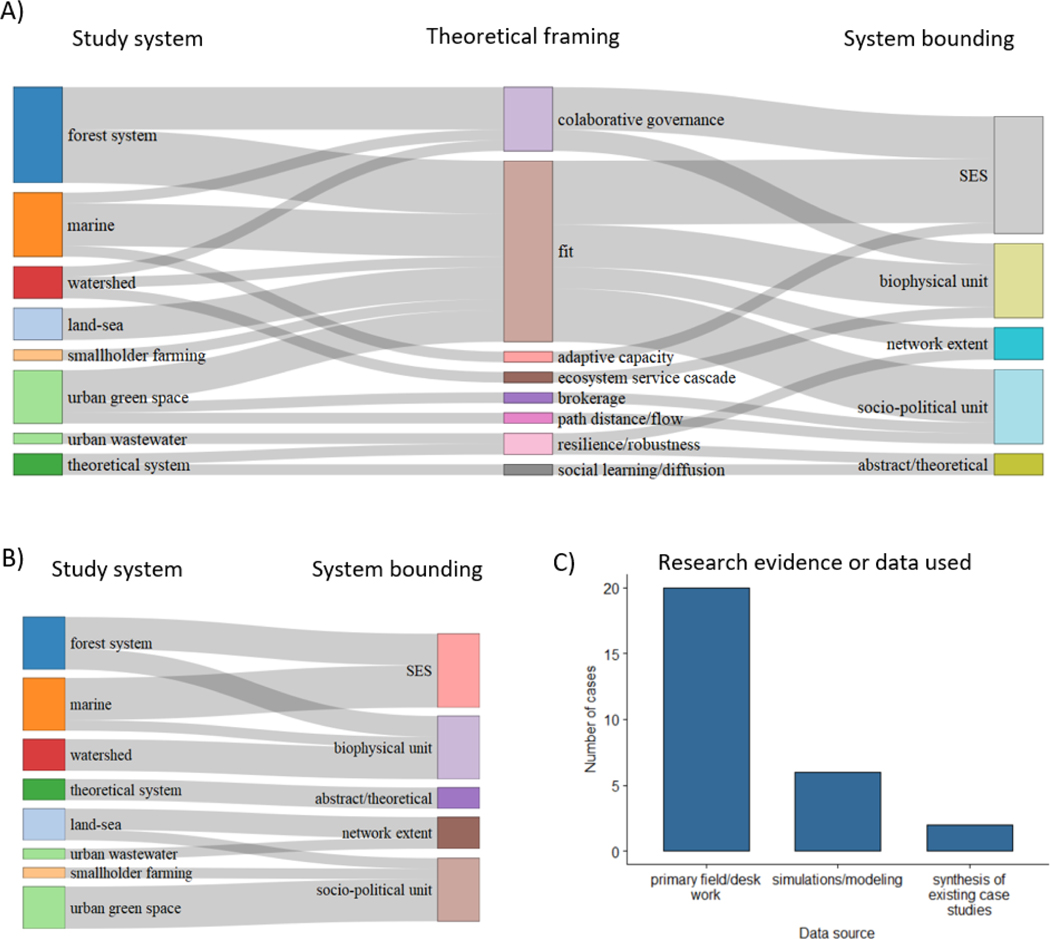
Relationships among A) chosen study systems, the theoretical issue addressed or how the paper was framed, and study system bounding (theoretical frames are defined in Table 4); and B) just study system and bounding to clearly illustrate several alignments among study systems and bounding. Because of multiple cases and multiple framings for some papers there are 30 paths in A on each side of diagram. In B, links equal the number of cases (n = 24). C) Bar chart of kind of evidence or data used in each case (n = 24). Several cases used multiple kinds of evidence.

The majority of fully articulated SEN papers address the problem of spatial or functional fit among social governance systems and the environment with a scattering of other theoretical framings (Fig. 5a, Table 4; focus on fit is also evident among the unifying citations in the citation network, SI). Empirical systems are far more diverse and include urban, forested, fluvial, and marine environments. Theoretical framings cross-cut the chosen study system and how the system is bound (Fig. 5a), while some bounding approaches align with the specific study system (Fig. 5b). For example, watershed and urban greenspace studies were bounded by biophysical and socio-political units, respectively. Finally, the majority of fully articulated SEN research is based on collecting primary data through field-based research and text analysis of grey and published literature (Fig. 5c).

To analyze these data and address objectives, papers used a number of analytical approaches. The most common involved motif frequency counts (e.g., Bodin and Tengö 2012, Kininmonth *et al* 2015, Bergsten *et al* 2019), which evaluate the relative prevalence of certain network structures (typically featuring a small number of nodes). This approach is conceptually similar to exponential random graph modeling (ERGM) – also a common approach in the studies we reviewed (e.g., Hamilton *et al* 2019, Guerrero *et al* 2015, Bodin *et al* 2016). ERGM evaluates the tendency for certain network structures to be over- or under-represented, but differ from motif frequency counts in several ways, such as in their ability to evaluate multiple structures at once and reliance on Markov chain Monte Carlo simulation. Other statistical modeling approaches included blockmodeling (e.g., Ekstrom and Crona 2017, Sayles and Baggio 2017a), and Quadratic Assignment Procedure (QAP) analysis (e.g., Alonso Roldan *et al* 2015, Bergsten *et al* 2014). A second group of studies utilized descriptive statistics (e.g., tabulating network metrics) or qualitative interpretation of networks as the primary analytical method (e.g., Zhao *et al* 2018, Ernstson *et al* 2010). In comparison to the statistical modeling approaches described above, these descriptive approaches principally served to facilitate exploratory analysis. These groups were not mutually exclusive; studies that relied on inferential modeling, for example, often included extensive descriptive analysis (e.g., Bergsten *et al* 2014). Finally, two studies used agent based or other computation modeling to analyze disturbances in social-ecological systems (Baggio and Hillis 2018, Chopra and Khanna 2014). While computational modeling was used less than the aforementioned approaches, some SEN research used simulations to generate network data that were subsequently analyzed using other statistical models (e.g., Hamilton *et al* 2019).

### 4.2. Network construction and conceptualization

The majority of papers conceptualized social nodes as some kind of collective social entity including organizations (n = 12, see SI) or other kinds of groups such as clans, fishers, and ecosystem service beneficiaries (n = 6). Social nodes rarely represented individuals (n = 2) and never represented households. Papers also included as social nodes, but to a lesser extent, undefined social actors in a model (n = 1), industries (n = 1) and DPSIR^7^ elements (n = 1). Ecological nodes were more evenly distributed among a range of concepts including groups of plants or animals (n = 4), habitat patches (n = 7), biophysical places (n = 6), and concepts of habitats and ecosystem types (n = 5). Several other categories were also considered (i.e., ecosystem services, sustainability issues, undefined, and DPSIR elements; n = 5, see SI). Individual plants or animals were never considered.

For the most part, social and ecological nodes were clearly differentiable, though several cases illustrate how node classification can be somewhat malleable. Ekstrom and Crona (2017) define nodes using the DPSIR framework and include a “fossil fuel” node in the category of “driver.” Here, the action of burning fossil fuel is a driver, but the stock of fossil fuel would likely be considered part of the biophysical subsystem. Likewise, two papers (Zhao *et al* 2018, Roldan *et al* 2015) included ecosystem services and beneficiaries. Based on the authors’ presentation, we classified these as ecological and social nodes, respectively; however, it might be equally possible to classify ecosystems services as a bridging node between distinct social and ecological nodes as proposed by Dee *et al* (2017).

Social edges were primarily nominal relationships (n = 17, see SI) and information or financial flows (n = 12), with several other considerations as well (i.e., productivity (n = 1), change in harvesting strategy (n = 1), and social influence (n = 1)). No study used social edges to represent issues like trust and legitimacy, which are fundamental to most, if not all, environmental governance issues. Nor did studies explicitly depict multiple edges to account for interplay among different kinds of social relationships. When multiple kinds of social relationships were included (e.g., collaboration and knowledge sharing (Guerrero *et al* 2015, Kininmonth *et al* 2015)), they were generally aggregated into a single relationship (though see Sayles and Baggio (2017a) for an example where one kind of social relationship was used to explain the outcome of another). These findings illustrate that more nuance could be brought into SEN analysis. Social-ecological systems often feature such “multiplex” linkages, which may encompass qualitatively distinct forms and types of social and social-ecological interactions (Baggio *et al* 2016, Schnegg 2018).

Ecological edges tended to represent the movement of plants and animals (n = 10, see SI), as well as concepts of ecosystem linkages (n = 7). Movement of physical materials (e.g., water or sediment) was considered to a lesser extent (n = 3). Animal trophic interactions and the concept of spreading ecological disturbances were each considered twice. Several studies aggregated multiple species, or biophysical processes, or both into a single presence/absence of an ecological relationship. Only one study considered dynamics among multiple ecological relationships (Ernstson *et al* 2010), which showed multiple ecological edges as descriptive model of the system, but did not analyze the interactions.

Social-ecological edges predominantly represented social entities acting or exerting agency on ecological entities (e.g., ownership/management (n = 17, see SI), harvest (n = 13), and human impacts/stressors (n = 4)).^8^ Several supporting/regulating relationships (n = 5), where ecological materials or functions flow to social entities regardless of active social agency, were also considered, as were several reciprocal relationships (n = 5). While many papers accounted for multiple social-ecological relationships (e.g., 45 edges among 24 cases), these were mostly aggregated into a single edge, or in the cases of the ecosystem service cascade (Zhao *et al* 2018), different kinds of social-ecological edges were considered among different types of nodes on a one to one basis. Interplay between different kinds of social-ecological edges was not considered.

Table 5 illustrates how the 22 fully articulated SEN papers adhere to different network models and approaches. Single-layer system, multiplex landscape, and multi-level landscape approaches were the most common. Pittman and Armitage (2017) and Bergsten *et al* (2019) represent somewhat of a hybrid between landscape and system approaches. Both analyze relationships among real and specific organizations connected to abstract environmental systems or issues, respectively. While ecological nodes and edges were theoretical, based on a general knowledge of the biophysical environments in question, organizations are linked to the environment based on actual management goals and mandates. Finally, Ernstson *et al* (2010) was classified as a multi-dimensional landscape model; however, the paper uses a narrative approach to synthesize and analyze several case studies. The authors build their narrative around a conceptual network model that can be considered multi-dimensional (they do not call it as such), but they do not employ multi-dimensional mathematics. Multi-dimensional networks are relatively underutilized in fully articulated SEN research.

## 5.0 Discussion

In the past decade, SEN research has transitioned from a theoretical proposal into a growing body of empirical studies of which we have focused on the “fully articulated” kind. By accounting for dynamics within and between social and ecological sub-networks, the fully articulated SEN approach holds great potential to understand human-environment systems by allowing researchers to hone in on the full spectrum of system interactions (Bodin and Tengö 2012). SEN research is nascent with ample room to grow, inform, and be informed by wider human-environment studies. The following offers guidance about research needs and opportunities, application of SENs to specific kinds of problems (including methodological and epistemological considerations), and when to use the SEN approach, fully articulated or otherwise.

### 5.1 What is the current scope of SEN research and what advancements are likely needed?

Fully articulated SEN research has been applied to a diverse range of study systems and contexts, demonstrating its popularity and applicability. Most SEN work has been applied and methodological, though some papers aimed to test theory. As with any emerging scholarship there is room to grow. Three important areas for growth include data and methods, enhancing causal inference, and further integration with key issues from wider human-environment research.

Many SEN studies to date have relied on primary field work for social data collection and coupled this with simpler presence/absence data of ecological relationships from existing data sets or theoretical understanding of the system. Future advancements may reside in collaborating with ecologists or geomorphologists to incorporate more nuanced environmental relationships (see the review by Turnbull *et al* (2018) for a detailed discussion of how these research communities use networks to understand their study phenomena). Fully articulated SEN research should also explore multiple relationships and how they interact, which might come from further work with multiplex and multidimensional networks approach (see section 5.2 for more details). There may also be fluidity among some of the social edge categories that we inventoried. As Guerrero *et al* (2015) note, activities like information exchange are likely implicit in all nominal relationships of collaboration. Still, information sharing and collaboration are not necessarily substitutable or reducible. Disentangling the many facets of complex relationships constructs, such as collaboration, may also be areas for future work.

Another area for future work will be to link SEN structures to environmental outcomes. We found that few studies concretely made this link, an observation that is consistent with broader social network studies on environmental issues (Barnes *et al* 2016, Groce *et al* 2018). If SEN research is to inform policy or practice, which many SEN studies aim to do, there needs to be a strong evidence base to back it. Many SEN studies have made diagnostic recommendations for management based on assessing, for example, spatial fit among social and ecological edges. The empirical evidence linking good alignment with successful outcomes is mixed however, according to the review by Bodin (2017). Of course, evidence may be mixed because there are very few causal examples and more testing may be needed. One challenge to linking structures and outcomes could be logistical. To date, SEN research has tended to focus on geographically large study areas and associated problems, settings where it can be hard to amass proper long-term datasets to document change over time. Indeed, only two studies have looked at multiple time periods (Zhao *et al* 2018, Yletyinen *et al* 2018). Integrating SEN research into nationally funded long-term research programs might be a way forward. In addition to amassing time series data, grouping case studies among core governance challenges and levels of abstraction, as proposed by Bodin *et al* (accepted), may be a way forward. Such a heuristic might help work through case by case idiosyncrasies to arrive at a more generalizable and causal understanding (Bodin *et al* accepted). Logistics and the lack of longitudinal data, however, may not be the only barrier. Differences in the time-scales at which activities such as collaboration and information sharing occur versus when ecological outcomes are observable, as well as myriad co-dependent factors, may also present barriers.

There is also significant room to engage other key human-environment issues, beyond fit and collaboration, two topics that have received a lot of attention to date. Several published commentaries have argued that network analysis can advance the study of ecosystem services (Bohan 2016, Bodin *et al* 2017, Dee *et al* 2017). We found limited engagement with ecosystem service frameworks beyond two diagnostic assessments of actor connectivity to improve ecosystem management (i.e., Zhao *et al* 2018, Roldan *et al* 2015). One area of future work will be, as Dee *et al* (2017) proposed, to relate specific social and ecological patterns (e.g., de/centralized governance and loss/restoration of key species) to ecosystem service delivery to test theories about what kinds of relationships support different ecosystem services (perhaps with a multilevel landscape or systems approach). Theory testing can guide ecosystem service policy and practice, and address the more general need to link network structure with social and ecological outcomes.

Fully articulated SEN approaches might also be integral to studying alternative perspectives in ecosystem service valuation. People often value specific places or specific ecosystem components, as opposed to abstractions. An old tree in a park where families picnic may be valued, whereas trees in general are not (Chan *et al* 2016). No fully articulated SEN research has worked with individual plants or animals as ecological nodes, which would be needed to study such alternative values specific to individual biophysical entities as opposed to environmental generalizations. Testing correlations among social values and ecological relationships (perhaps with a multiplex landscape approach) might provide bridging concepts to help communicate the benefits of ecosystem-based management. This work also has relevance for sense of place and place-making (which could use a multi-level landscape approach). For example, how do people value the places connected to the places they value? How does creating new ecological or social relationships change these values? Data about the strength of environmental links and social values could bring a high level of nuance to such analyses and help advance SEN methodology as most studies to date have investigated presence/absence of relationships.

Network science concepts and tools have also been highlighted among the telecoupling research community (Eakin *et al* 2014, Liu *et al* 2015). Telecoupling studies how two or more places that are considered, and therefore governed, to be independent are in fact connected and impact each other through material, market, or information flows. Additional places not directly involved in the exchange may also be affected through spillovers (Liu *et al* 2013, Eakin *et al* 2014). We found no engagement with telecoupling frameworks, revealing another area for synergy. The potential to represent telecoupled systems as networks should be immediately apparent. For example, Liu *et al* (2013) have questioned how one-to-one versus one-to-many relationships among sending, receiving, and spillover systems shape the processes and outcomes of telecoupling. These structural arrangements can be easily studied using multi-level system or landscape approaches. Network synthesis efforts, such as Barnes *et al’s* (2017) work on adaptation and transformation, illustrate how analysis of network structures can inform human-environment theories. Similar synthesis work could help unpack big telecoupling questions such as why one place is able to exert influence over another or is vulnerable to spillovers (Liu *et al* 2013).

Telecoupling and ecosystem services represent high profile research areas that have looked to the network sciences, but in no way are they the only areas for fruitful advancements. Scholars should look to any number of key issues germane to the human-environment sciences. Interesting opportunities may also lie in somewhat less talked about research areas such as relational and process-based philosophy. In this ontology, the world consists of relations, not objects (Cooke *et al* 2016, West *et al* 2018, Kaaronen 2018, Mancilla Garcia *et al* 2019). Such an ontology, for example, argues that valleys and mountains only exist in relation to each other. Neither is an individual object, as descending down a mountain or into a valley is an artificial bifurcation. Instead, this perspective argues that movement is real, and it is how we experience the world (Ingold 2006). In such an ontology, people and nature cease to be discreet objects, but rather a single, integrated human-environment web (Debaise 2017). SEN approaches add a layer of thinking and methodology to help grasp this ontology. How, for example, do the number, kinds, and configuration of relationships between people and non-humans, or places, shape values and behaviors? Process philosophy puts specific emphasis on human-nature unity (Kaaronen 2018), what is often referred to as co-produced phenomena, products of human and non-human processes (Turner 2002, West *et al* 2018). This focus could introduce a third kind of node to fully articulated SENs, a co-produced node, which is not reducible to the social or ecological subsystem. Indeed, this is what Dee *et al* (2017) propose for ecosystem services research. Co-produced nodes might expand our definition of fully articulated SENs, or perhaps create a new SEN category, though it is probably not worth focusing on the semantics of classification. Attention should be directed at how to better understand human-environment processes and outcomes.

A key challenge when integrating SEN concepts with other human-environment framings (which are not limited to those discussed above) will be to focus on what unique conceptual and theoretical contributions SEN research offers. Hamilton *et al’s* (2019) study of wildfire risk, for example, illustrates how network concepts of structure and position can be used to develop theories about social-ecological systems. Hamilton *et al* (2019) developed and tested hypotheses about why land managers coordinate to mitigate wildfire risk; coordination was hypothesized to vary as a function of its costs and benefits, which in turn depended on burn patterns and positions of land managers in a SEN. If SEN research is to advance, it cannot be reduced to a methodology for other sub-disciplines or framings. A phenomenon of study, not methods, define a (sub)discipline (Turner 2002).There must be a true melding of theories and approaches from the network sciences and other human-environment efforts.

### 5.2 What fully articulated SEN models seem best for different environmental research issues; and what are some methodological and epistemological implications?

The fully articulated SEN papers in this review represent a diverse range of approaches to conceptualizing and analyzing social-ecological systems. Two fundamental aspects of any network study are how to model and bound a study system. Addressing these aspects undoubtedly affects how the studied phenomena is conceptualized and quite possibly the kinds of results that can be deduced.

Model approach (Table 5) affects how the world can be represented in terms of the number and kinds of entities studied, and how they can be related. Based on the examples documented in this review, single-layer models are most commonly used to analyze a social-ecological system in abstraction. Hypothetical or generalized user groups, environmental components, or system processes can be related to each other in various ways. While often used to address generalized and abstract entities or processes, detailed analysis about specific actors can still be achieved. Ekstrom and Crona (2017), for example, look at responsibility among US federal agencies for dealing with ocean acidification and are able to recommend specific needed partnerships. The network provides a general systems framework for organizing federal agency and environmental interactions. The network analysis reveals coarse grained patterns that can then be used to hone in on specific dynamics, which can be investigated in more detail.

Representing detailed and specific places or organization tends to require more complex models such as multiplex and multi-level. Still, in three of the multi-level landscape models that we documented (Bergsten *et al* 2014, Treml *et al* 2015, Hamilton *et al* 2019), authors aggregated and generalized ecosystem structure to “scale it up” to larger socio-political entities. Jurisdictional units were used to define a common place-based node among an ecological and a social layer that were conceptually linked by management responsibility. A high degree of environmental specificity is lost in this aggregation, which might limit applied utility for managers or planners working at a local level. Still, general patterns are revealed and authors are able to test theories such as what explains municipal collaborations: proximity or ecological connectivity (i.e., Bergsten *et al* 2014). While the multiplex model requires a common node among social and ecological network layers, it is not necessary to aggregate up to the larger unit, socio-political in the documented cases. (There is also no reason why studies cannot involve more than two layers.) While we did not find examples that disaggregated larger spatial units, such as jurisdictions, to smaller ones, such as small habitat patches, it is theoretically possible. The pros and cons of scaling up or down remain a little unclear, with the exception that results at a given resolution will likely resonate with a given audience, such as local versus regional planners, more than others. SEN research might benefit from a series of methodological studies that experiment with aggregating and disaggregating social and ecological entities that are of different geographic sizes to provide better guidance on how such decisions affect study outcomes.

To link individual organizations to specific habitat patches or biophysical areas, authors used multi-level models, which have been a favored approach. All multi-level landscape cases looked at patterns among small sets of nodes and their edges to analyze the SEN, often through ERGMs, which provide global level statistics, such as identifying which specific patterns explain how the network is shaped. Global level results are quite useful for hypothesis testing, such as identifying if patterns associated with good fit are present in successful resource management cases (Bodin and Tengö 2012, Bodin *et al* 2014). Global results may not provide detailed information about specific actors or places, however, which might inform policy and management actions. Such actor, place, and issue specific information has been derived from SENs using ERGM node counts (Bodin *et al* 2014), block modeling (Sayles and Baggio 2017a), and node-positional analysis (Bergsten *et al* 2019).

Given the popularity of multi-level networks among our documented examples, it is tempting to conclude that they are the preferred approach for fully articulated SEN analysis. Multi-level networks are likely attractive because they allow two or more kinds of nodes, whereas multiplex models require translating social actors and ecological structures into some kind of places-based node (for landscape models) that can hold the attribute of both social and ecological entities. Operationalizing the world as a SEN is slightly limited in multiplex models. Still, the multiplex networks are probably underutilized (Baggio *et al* 2016). Researchers can infer correlations and patterns among all nodes and edges in the SEN simultaneously (Boccaletti *et al* 2014) as opposed to reducing the network into smaller subsets of nodes and edges, which is often done for multilevel analysis. Some of the greatest conceptual and methodological advances may be realized with multi-dimensional models, which have yet to be truly employed among SEN research. These models allow multiple kinds of nodes with multiple edges.

Study bounding is the other major area that shapes problem formulation. The fully articulated SEN studies reviewed here used a variety of criteria to bound study systems such as biophysical areas, socio-political units, or tracing specific social and ecological entities to bound the study based on the social-ecological system in question. Any bounding will undoubtedly be an artificial cut because social and ecological entities always have linkages that extend beyond a given study system. For example, Sayles and Baggio’s (2017a) study focused on restoration in the Whidbey Basin watershed and used the watershed to bound the system. Further research by Sayles (2018) shows how actors and biophysical dynamics outside the Whidbey Basin watershed affect, and are affected by, happenings within the watershed. Following the network and letting it define the boundary is no less immune to such challenges. All possible related issues and relationships simply cannot be accounted for in a study. Bounding will affect the kind and number of nodes and edges included, and network analysis is particularly sensitive to changing these numbers. While there will never be a single answer for study bounding, SEN research may again benefit from comparative methodological studies on the effects of bounding approaches.

### 5.3 When to SEN?

Our review has focused on empirical studies of fully articulated SENs, which account for connectivity within and between social and ecological nodes. While this perspective allows researchers to analyze diverse forms of connectivity in social-ecological systems, such an approach is not always appropriate. Empirical, theoretical, and logistical considerations may each limit the value or viability of the fully articulated SEN approach. For example, Yu *et al* (2014) use a partially articulated SEN approach to study how globalization induces transformation in common pool resource management. They predominantly rely on historical archives and are only able to document social-ecological edges in their study. Data on inter-forest patch dynamics and intercommunity relations were not available. The authors are still able to address their research questions and illustrate how several network properties affected the transformation of the forest commons.

Likewise, a non- or partially articulated SEN perspective may better fit the research questions at hand. Rathwell and Peterson (2012) are able to assess how municipal collaborations relate to landscape management choices and water management activities by looking at relationships among social actors (social edges) and where they work (social to ecological edges). While assessing ecological relationships such as hydrology or green corridor connectivity (ecological edges) might have allowed them to answer further questions, such ecological relationships are not fundamental to answer their original research questions.

While there is much to gain from the fully articulated SEN approach, it should be seen as one of many approaches to understand social-ecological systems, guide management and policy, and test theories. For example, while a large number of fully articulated SEN studies contribute to the theory of institutional fit (Fig 3) -- possibly because the theory identifies hypotheses that readily translate into structural relationships between social and ecological systems -- studies that adopt less articulated SEN conceptualizations have also advanced the theory of institutional fit. Alexander *et al’s* (2017) study of marine protected area (MPA) governance did not explicitly measure connectivity among MPAs, but nevertheless improved understanding about how patterns of collaborations among stakeholders can increase social-ecological fit.

Collecting data about social and ecological interactions often requires considerable investment, especially when relying on primary data, as was evident in the majority of published fully articulated SEN studies. When such an approach serves more to contribute contextual richness than to help address research questions, researchers may elect to prioritize certain subsets of linkages rather than collect data needed to describe a fully articulated SEN. This guidance applies equally to non- and partially articulated SENs. Networks are a powerful way to view and analyze the world. They capture the imagination and often render fascinating maps. Not all research questions are network questions, however. If a question is not rooted in relationships, it might be better served by other research approaches.

## 6.0 Conclusion

The SEN approach has immense potential to help understand social-ecological systems and address environmental problems. What we have termed “fully articulated SENs” provide a particularly attractive approach because diverse social, ecological, and coupled relationships can be represented and analyzed. Translating the world into a network for analysis requires certain assumptions. The approaches and models outlined above can help those new to SEN research determine if SENs are the right approach (i.e., “When to SEN?”) and if so, how best to work with SENs.

In the past half-decade, there has been significant development of fully articulated SEN ideas and applications, with the qualification, of course, that the current pool of substantive studies is small (but growing). Potential future applications and advancements are promising and ample as outlined throughout this review. How these advancements take shape may potentially move SEN research beyond a way of thinking and set of methods into something more, though we refrain from speculating exactly what that may be. Amassing more case studies that firmly link SEN structure to outcomes would provide an evidence base to test theories, perhaps develop new ones rooted in network structure, and guide environmental management.

The quest for inference, however, should not overshadow the demonstrated and potential strength of SEN research to diagnose conditions that (proven or theorized) enhance environmental governance and management. Under this applied research trajectory, scholars may wish to explore how best to communicate SEN concepts and ideas, or how to put network science tools in the hands of practitioners and stakeholders. Through research and professional appointments, many of the authors here have observed that the practitioner community is often enthusiastic about networks, but sometimes lacks exposure beyond activities such as stakeholder mapping. While anecdotal, this evidence suggests that SEN scholars might do more to transfer and translate the full richness of network ideas and methods to those working in and managing the systems that we all study. Transdisciplinary collaborations may be an important step to advance the applied and policy theme that, as we documented, runs strong within fully articulated SEN research.

## Supplementary Material

SI

## Figures and Tables

**Table 1.** Coding scheme to address how fully articulated SEN studies have been framed and conducted. Methods are abbreviated as follows: CC = close-ended categorical, OC = open-ended categorical, MR = multiple responses permitted, SR = single response only, OT = “other” write-in option possible. Details in SI.

Variable	Method	Options and definition (if warranted)
Analytical approach	CC, MR	**Diagnostic approach**: *identify good or bad structures for a given objective* **Inferential approach** ·**Network as outcome**: *test or explain what shapes the network* ·**Network as explanatory**: *use network to test or explain a given outcome*
Authors’ stated objectives	CC, MR	**Testing theory** **Applied or policy focused** **Methodological advancement**
Study system	OC, SR	*Responses were inductively classified into categories*
Theoretical framing	OC, MR	*Responses were inductively classified into categories*
System bounding	CC, OT, SR	**Socio-political unit**: *e.g., a given municipal border.* **Biophysical unit**: *e.g., a watershed* **Network extent**: *see note A* **Abstract/theoretical**: from *write-in; see note B* **Social-ecological-system**: *from write in; see note C*
Evidence used	CC, OT, MR	**Field/desk work**: *empirical investigation through field work or “desk methods”; e.g., document coding* **Simulation or modeling** **Synthesis of existing published case studies** *(No alternative write-ins recoded)*
Methods used	OC, MR	*Responses were inductively classified into categories*

Notes: A) Network extent refers to cases were researchers, once having identified a logical social and/or ecological starting point followed the network until its logical end (for example, from a social network methodological perspective, this would most likely be done using “snowball” sampling). B) Some modeling studies were based on a theoretical universe and thus, had an abstract or theoretical bounding. C) Several studies were bound by the social-ecological system. In this case, not all social or all ecological units in a given arena are included in the network, but rather specific actors, organizations, or institutions were selected alongside corresponding resource units, habitat patches, or other environmental areas based on an *a priori* detailed understanding of the social-ecological system.

**Table 2.** Coding scheme to address how fully articulated SENs are constructed. Abbreviations are defined in Table 1; IT = iterative process. Details in SI.

Variable	Method	Options and definition (if warranted)
Social nodes	CC, OT, MR	**Individuals** **Households** **Organizations** **Policies / Laws** **Human management actions or stressors** **Other** *(several reported; see* section 4.2*)*
Ecological nodes	CC, OT, MR	**Individual plants/animals** **Groups of plants/animals** **Specific habitat patches:** *spatially discrete, e.g., wetland or forest patches* **Biophysical places/areas:** *contiguous, e.g., watersheds, marine areas* **Concept of habitats/ecosystems** **Other**: *(several reported; see* section 4.2*)*
Social-social edges	CC, OT, MR	**Nominal:** *defined simply as having a relationship; e.g., collaborators, co-signers of a policy or law* **Flow:** *defined through exchange; e.g., knowledge/resource sharing, learning, funding* **Measures of performance:** *e.g., productivity or self-reported importance for achieving goals* **Measures of trust or legitimacy** **Other:** *(several reported; see* section 4.2*)*
Ecological-ecological edges	CC, OT, MR	**Movement of plants/animals** **Movement of water, sediment, or biophysical materials** **Trophic interaction** **Concepts of ecosystem / environmental linkages** **Other:** *(several reported; see* section 4.2*)*
Social-ecological edges	CC, OT, MR	**Ownership/management:** *social node managing, working in, owning, etc. the ecological node.* **Harvest:** *social node harvesting or actively getting something from the ecological node. Relationship would not exist without action by social node* **Supporting/regulating:** *flow of ecological process back to social node independent* *of the social node’s activity (though social node must be in spatial or power relationship that allows benefit); e.g., storm protection, carbon sequestration.* **Reciprocal:** *co-produced and cannot be reduced to social acting on ecological, or ecological flowing to social without social agency; e.g., intrinsic or spiritual value, recreation. See note.* **Other:** *(several reported; see* section 4.2*)*
Network conceptualization	IT	*Papers were classified based on typologies in* section 2.2. *This was arrived at iteratively during the analysis of the papers leading to a posteriori classification.*

Note: While arguably all social-ecological relationships are co-produced, the distinction here is on the dominant direction of agency or flow in creating the relationship.

**Table 3.** List of 22 identified SEN papers sorted by year and alphabetically. IDs indicate papers in the network diagram, Fig. 3.

SEN Papers (1–11)	ID in Fig. 3	SEN Papers (12–22)	ID in Fig. 3
Ekstrom and Young (2009)	59	Bodin *et al* (2016)	24
Ernstson *et al* (2010)	63	Dragicevic and Shogren (2017)	53
Bodin and Tengö (2012)	28	Ekstrom and Crona (2017)	56
Bergsten *et al* (2014)	12	Pittman and Armitage (2017)	123
Bodin *et al* (2014)	23	Sayles and Baggio (2017a)	134
Chopra and Khanna (2014)	37	Xiu *et al* (2017)	156
Guerrero *et al* (2015)	76	Baggio and Hillis (2018)	6
Kininmonth *et al* (2015)	90	Yletyinen *et al* (2018)	158
Prager and Pfeifer (2015)	124	Zhao *et al* (2018)	163
Roldan *et al* (2015)	130	Bergsten *et al* (2019)	13
Treml *et al* (2015)	143	Hamilton *et al* (2019)	80

**Table 4.** Definitions and descriptions of theoretical framing used in the documented SEN papers. Categories in column one represent how authors presented their research and are not an *a priori* classification. In trying to adhere closely to authors’ own depictions of their work, the categories are not always of the same magnitude or complexity, nor are they meant to be. Collaborative governance and fit, for example, are broader concepts than brokerage.

Theoretical framing (number of papers)	Definition and Description
Collaborative governance (n = 6)	**Definition:** Multiple actors, often with different interests, working in different places and/or at different administrative levels, interacting in one or more ways to set rules and norms for governing the environment (Bodin 2017). **Description:** Papers focused on collaboration and cooperation, in which actors collectively solve problems, as well collective action, which addresses the tradeoff of collective rewards and individual costs for actors engaged in environmental activities.
Fit (n = 17)	**Definition:** How rules, norms, and approaches to govern environmental problems align in one or more ways with those problems and their context. Epstein *et al* (2015) distinguish three types of fit: aligning with the biophysical system (ecological fit), social system (social fit), or their interplay (social-ecological systems, or SES, fit). **Description:** Papers addressed ecological fit. They looked at geographic alignment between social and ecological networks (spatial ecological fit), or how well institutions and policies addressed ecosystem dynamics (functional ecological fit). Broader social-ecological dynamics were often considered, thus also engaging SES fit.
Adaptive capacity (n = 1)	**Definition:** The ability to reduce exposure or sensitivity to disturbances, usually by shifting or changing rules, norms, behaviors, or activities (Gallopin 2006). Adaptive capacity is strongly related to the concept of resilience (below); however, to reflect how authors described their work, we separated these concepts. **Description:** The paper focused on the how changes in harvesting strategies affect resource dependence and exposure.
Ecosystem service cascade (n = 1)	**Definition:** A framework recognizing how the benefits people derive from nature (i.e., ecosystem services) start with the production of benefits and end when beneficiaries are in spatial, temporal, and socio-political positions to access them (de Groot *et al* 2010). **Description:** The paper operationalized the definition as described above.
Brokerage (n = 1)	**Definition:** How key actors mediate social relations among disconnected actors or groups, through bridging or bonding social capital (Burt 2005). **Description:** The paper focused on urban wildlife management among places and administrative levels.
Path distance and flow (graph theory) (n = 1)	**Definition:** Paths are the number of unique nodes visited when moving between two nodes in a network. Flow represents the cost or resistance of moving along an edge. Calculating minimum and maximum paths, often weighted by flow, is part of graph theory, the body of mathematics that describes networks (Urban and Keitt 2001). **Description:** The paper focused on balancing human recreation and wildlife needs.
Resilience and robustness (n = 2)	**Definition:** Related concepts (though not synonymous) describing a system’s ability to maintain identity and function when disturbed. A resilient system may also change and redevelop when it can no longer absorb disturbance (Anderies *et al* 2004, Folke 2006). **Description:** Papers studied how changing specific nodes affected network function.
Social learning and diffusion (n = 1)	**Definition:** How knowledge is exchanged and its co-dependence with environmental disturbances; see references in Baggio and Hillis (2018). **Description:** The paper operationalized the definition as described above.

**Table 5.** Fully articulated SEN study classification among four dominant network models and two general approaches. Models and approaches are described in section 2.2.

Network model	“Landscape” approach Geographically and/or spatially defined. Generally represent real-world entities, but could be modeled.	“Systems” approach A-spatial / abstract (though based on real world) or theoretical system.
**Single-layer** One layer of nodes with one edge possible between nodes. Can specify social/ecological using attributes.	Prager and Pfeifer 2015 Roldán *et al* 2015	Chopra and Khanna 2015 Ekstrom and Crona 2017 Ekstrom and Young 2009 Zhao *et al* 2018
**Multiplex** Allows multiple kinds of edges, each a different network layer. All nodes must be present in each layer.	Baggio and Hillis 2018 Bergsten *et al* 2014 Hamilton *et al* 2019 Tremel *et al* 2015 Xiu *et al* 2017	Dragicevic and Shogren 2017
**Multi-level** Allow different kinds and numbers of nodes in different layers. Edges are within and between layers, but one kind each.	Bergsten *et al* 2019* Bodin and Tengö 2012 Bodin *et al* 2014 Bodin *et al* 2016 Guerrero *et al* 2015 Kinninmonth *et al* 2015 Pittman and Armitage 2017* Sayles and Baggio 2017	Bergsten *et al* 2019 * Pittman and Armitage 2017 * Yletyinen *et al* 2018
**Multi-dimensional** Allows different kinds and numbers of nodes in different layers and multiple edges within and among different node types.	Ernstson *et al* 2010 **	no current examples

* Blends landscape and systems approach;

** Qualitative analysis built around conceptual network model.

## Footnotes

1 We use the term “human-environment” to refer to the broad collection of interdisciplinary research traditions that address coupled human-environment or social-ecological systems. We consider network scientists discussed in this review to be part of this wider research community. When discussing specific objects of study, we used the term “social-ecological system” to be consistent with the dominant literature doing SEN research.

2 Several previous reviews have also focused on social network analysis for natural resource governance (i.e., Bodin and Crona 2009, Bodin and Prell 2011, Groce *et al* 2018), but these only focus on social network studies that deal with natural resource issues; the network under investigation is only the social system, not a SEN.

3 Sometimes the term vertices or actors is used instead of nodes, and links, arcs (only when edges are directed) or ties are used instead of edges.

4 While this layered depiction is apt for conceptualizing multiplex models, they can be represented mathematically in several different ways and the social-ecological edge can (though not always) be defined mathematically as an adjacency tensor, as opposed to simple inter-layer alignment (Kivelä *et al* 2014, Baggio and Hillis 2018). There are also examples in which slight differences in the number of nodes can be permitted between layers (Kivelä *et al* 2014), though for conceptualizing SEN approaches, we stick to the more traditional depiction where the same nodes are present in each layer. Multiplex networks are sometimes called multirelational networks (Kivelä *et al* 2014).

5 Multiplex, multi-level, and multidimensional networks all belong to a common family of multi-layer networks. See the review by Kivelä *et al* (2014).

6 “Coupled human and natural systems” are often abbreviated as “CHANS.”

7 Driver, Pressure, State, Impact, Response framework.

8 Direction of influence reflects how the edges were conceptualized. Direction here does not mean that the networks used a mathematical network approach called “a directed network,” which analyzes if relationships go from node A to B, or B to A, or both. Networks can also be “undirected,” meaning a relationship between A and B does not distinguish any relationship direction (e.g., information is shared among A and B, but the flow direction is not specified).
