# Supplementary material for: Social-ecological network analysis for sustainability sciences: a systematic review and innovative research agenda for the future
Source: Environ Res Lett. Author manuscript; Available in PMC 2022 Mar 24. (PMC8943837; doi:10.1088/1748-9326/ab2619)
Supplement: SI [file NIHMS1539505-supplement-SI.pdf]

Supplemental information

SI. 1 Flow diagram of systematic review inclusion criteria

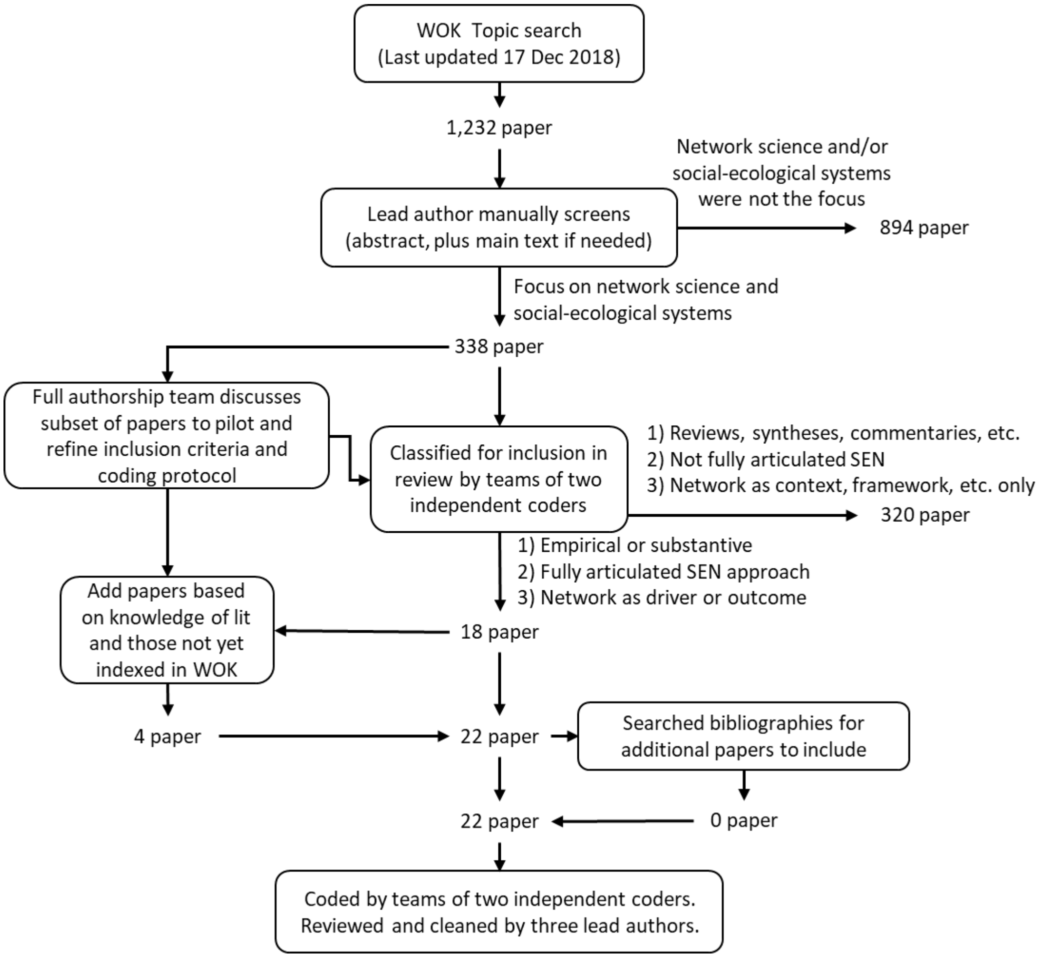

Figure S1. Flow diagram of systematic review inclusion criteria

SI.2 Details of attribute coding

To understand our first line of inquiry, how fully articulated SEN studies have been framed and conducted, we coded the papers' objectives, theoretical framing, how the study was bound, and the kinds of evidence and methods used. We considered objectives from two perspectives. We first coded if the paper took a diagnostic or inferential approach to analysis. Many network studies identify good or bad structures for a given outcome based on theory, i.e., a diagnostic approach. An alternative is to take an inferential approach and try to test or explain what shapes the network (network as outcome) or how the network explains a given outcome (network as explanatory). We also coded authors' stated objectives as testing theory, applied or policy focused, or methodological advancement. Papers could have more than one approach and objective. Study systems and theoretical framings were open coded and then inductively fit into categories. System bounding was categorized as socio-political unit, where for example, the study universe was bounded by a given municipal border; biophysical unit, such as a watershed or marine system;

network extent, in which researchers, once having identified a logical social and/or ecological starting point followed the network until its logical end (from a social network methodological perspective, this would most likely be done using "snowball" sampling, for example); or "other." Through the last category, two additional bounding approaches emerged: some modeling studies were based on a theoretical universe and thus, had an abstract or theoretical bounding. Several studies also were bound by what we call the social-ecological system. In this last case, not all social or all ecological units in a given arena are included in the network, but rather specific actors, organizations, or institutions were selected alongside corresponding resource units, habitat patches, or other environmental areas based on an *a priori* detailed understanding of the social-ecological system. Finally, the kind of evidence used in the paper included empirical investigation though field work or "desk methods" (e.g., document coding), simulation and modeling work, synthesis of existing published case studies, or "other" of which no alternatives were identified. Methods were open coded and inductively fit into categories. All variables allowed for multiple coded responses, except for study system and system bounding.

Our second line of inquiry, how fully articulated SENs are constructed, focused on the kinds of nodes and edges in the network and how the networks were conceptualized according to section 2.2. We categorized social nodes to illustrate different kinds of social actors or phenomena including individuals, households, and organizations, as the choice to focus on individual versus collective entities (e.g., organizations) impacts what can be learned about the underlying social dynamics (Butts 2009, Newig *et al* 2010, Sayles and Baggio 2017b). We also considered policies/laws and human management actions as other social entities that are often represented in SENs (e.g., Ekstrom and Young 2009) and allowed for additional write-in responses. Ecological nodes were classified as individual plant/animals, groups of plant/animals, specific habitat patches, biophysical places/areas, concepts of habitats/ecosystems, plus the option of an "other" write-in response. These categories capture a wide range of ways that researchers might represent the environment and were also informed by experiences that some physical phenomena more readily translated into the concepts of nodes. For example, small discrete forest patches or wetlands naturally form a network, whereas other biophysical phenomena, like surface hydrology or forest fires, are a more contiguous biophysical surface and require different assumptions to translate into nodes and edges (Sayles and Baggio 2017a, Turnbull *et al* 2018, Hamilton *et al* 2019). These might be seen as biophysical places or areas.

We categorized social edges based on the general type of relationship they represented. Following Borgatti *et al* (2009), we distinguished between nominal relationships, those representing social roles such as friends, partners, or collaborators, and those representing flows such as information, financial, or resources sharing. We included measures of performance as a unique category given the importance of outcome metrics for advancing environmental network research (Barnes *et al* 2016, Groce *et al* 2019), as well as a category on concepts of trust and legitimacy as these are important drivers of institutional structure (Berardo and Scholz 2010, Lubell *et al* 2014). Other write-in responses were permitted. Ecological edges were classified as movement of plants and animals, movement of water, sediment, or biophysical materials, trophic interactions, concepts of ecosystem / environmental linkages, and allowed for an "other" write-in response. These codes illustrate the different ways authors might depict ecological interactions

as both real and conceptual systems and in discrete or contiguous landscapes (Sayles and Baggio 2017a, Turnbull *et al* 2018, Hamilton *et al* 2019).

Finally, we defined social-ecological edges in a similar manner to how social edges were defined, by focusing on the character of relationships and interactions embodied by the edge. We considered relationships of ownership or management, which are similar to the social category of nominal, where the relationship between the social and ecological node was defined based on the social node having management jurisdiction or working in a given ecological area. We then considered different types of agency and flow. Harvest relationships described relationships that would not exist without action by social nodes (e.g., harvest or extraction). Supporting/regulating relationships described the flow of ecological processes back to the social node independent of the social node's activity (e.g., storm protection or carbon sequestration), though we acknowledge and recognize that the social node must be in a spatial or power relationship, or both, that allows for benefits. Reciprocal relationships described co-produced interactions that cannot be reduced to the social node acting on the ecological, or the ecological flowing to the social without social agency. While arguably all social-ecological relationships are co-produced, the distinction here is on the dominant direction of agency or flow in creating the relationship. For example, there is a categorical distinction between something like resource extraction (harvest category) and spiritual value, sense of place, and recreational fulfillment (reciprocal category). Of course, the two are not mutually exclusive; someone can find spiritual value through their resource extraction (and the edge would be coded as representing two phenomena). Other write-in responses were also permitted. All node and edge variables allowed for multiple coded responses.

SI.3 Inventory of nodes and edges used to construct SENs

The pairing of social and ecological nodes and edges illustrates how authors have constructed SEN. The following figures present the count of specific node and edge pairings.

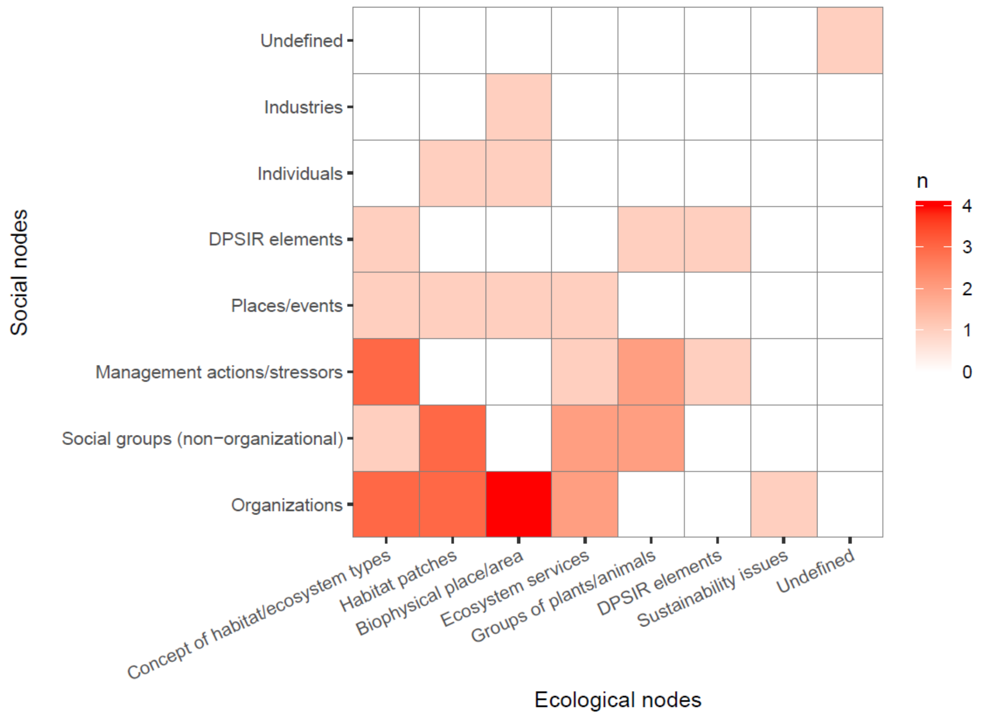

Figure S2. Frequency of social and ecological nodes pairings in 24 SEN case studies

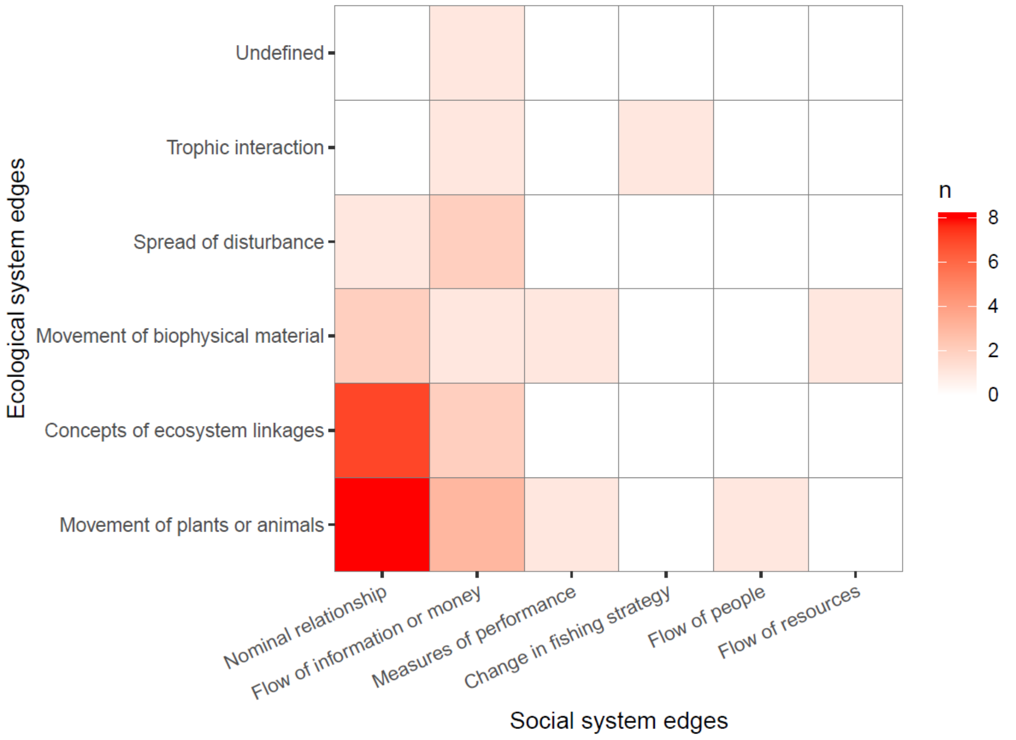

Figure S3. Frequency of ecological and social edge pairings in 24 SEN case studies

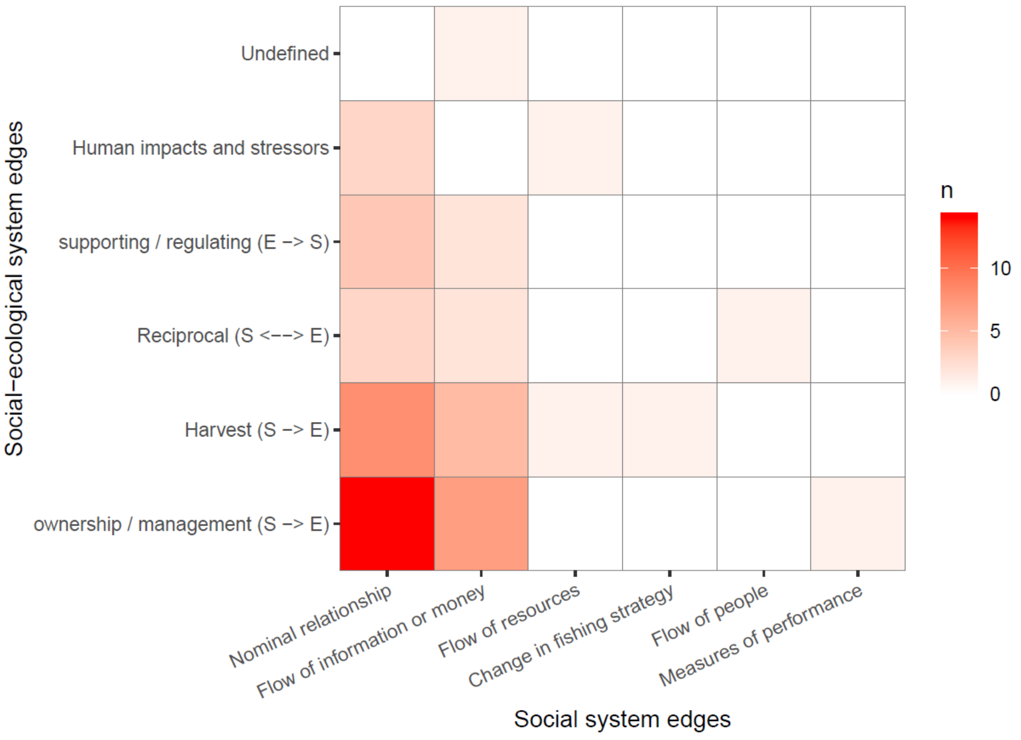

Figure S4. Frequency of social-ecological and social edge pairings in 24 SEN case studies

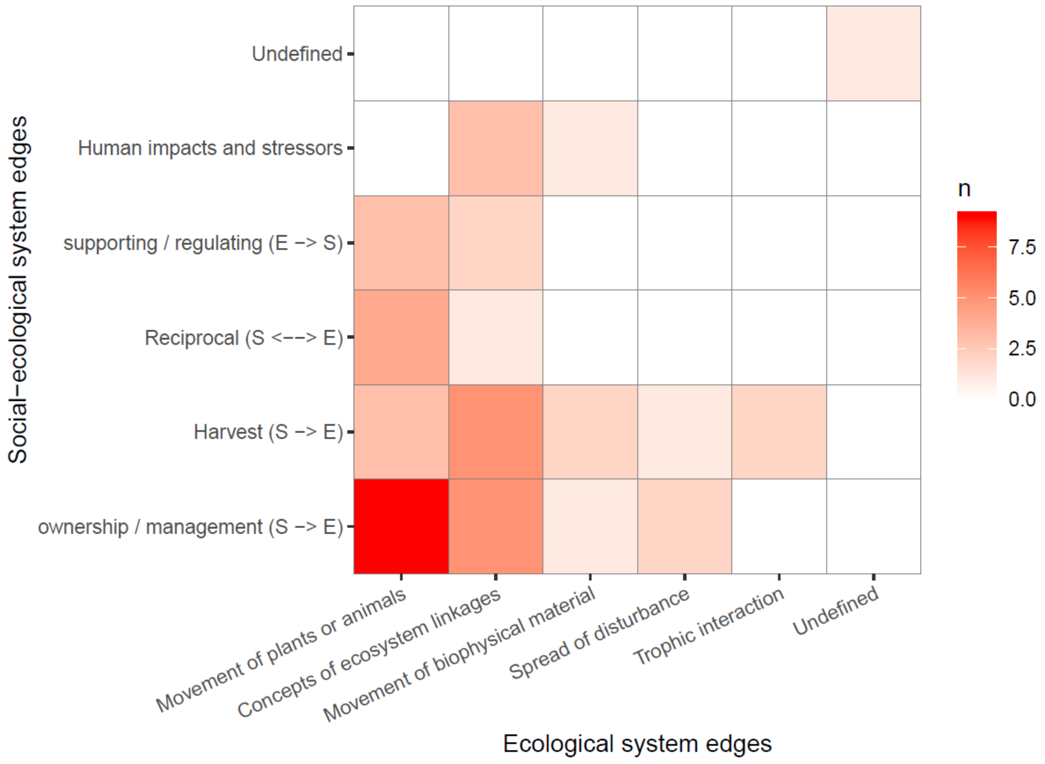

Figure S5. Frequency of social-ecological and ecological edge pairings in 24 SEN case studies

SI.4 Details of the citation network analysis

To understand the cohesiveness of fully articulated SEN research we conducted a citation network analysis. We did not expect all papers to be linked through direct citations, but did expect papers to draw from common theoretical works. We therefore constructed a bibliometric network of fully articulated SEN papers and their cited references to look at their common intellectual roots. We reduced the network to consider only cited papers with at least two citations, omitting citations that did not bind the network together. We used Freeman's degree centrality, which considers the number of cited references (fully articulated SEN papers only) and citations (for all papers). Only considering citation count (i.e., indegree centrality) would omit new SEN papers (e.g., Bergsten *et al* 2019, Hamilton *et al* 2019) that had not yet accrued citations. We then analyzed citation patterns using indegree centrality to understand which papers were common among SEN articles. Analysis was done in the R language environment using the packages network and sna (Butts 2015, 2016). The following figures show the full citation network prior to reduction by Freeman's degree centrality, the reduced network with all nodes labeled by IDs, and corresponding table of 22 fully articulated SEN papers and common cited references.

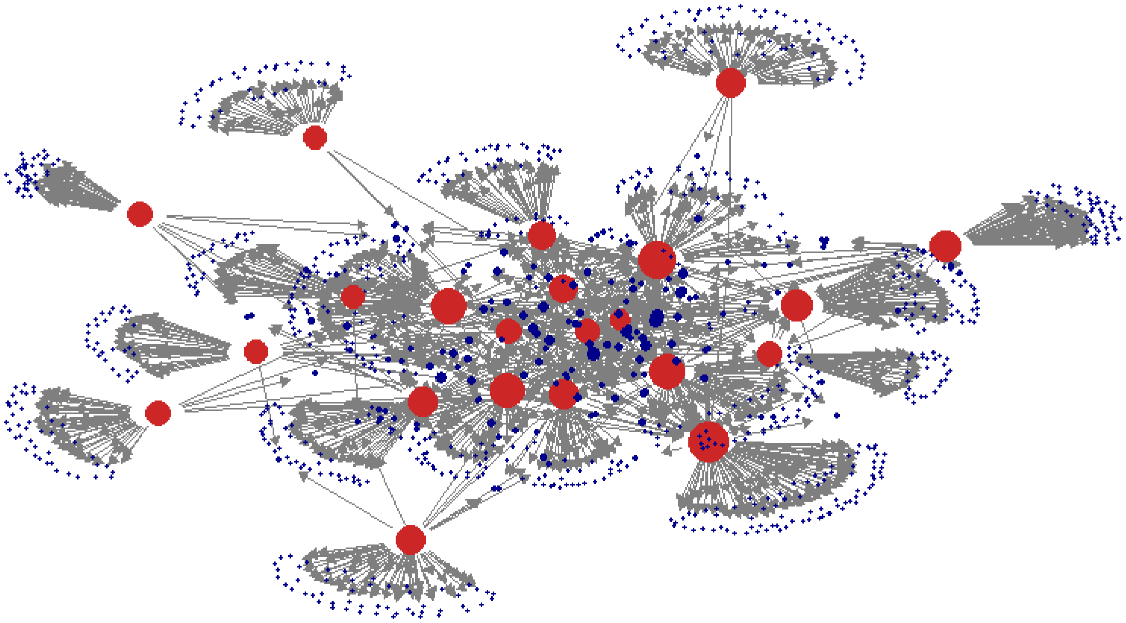

Fig S6. Network diagram showing the 22 SEN papers (red) and all cited papers (blue, n = 995; total network = 1,017 nodes). Node size represents the total number of cited references (for 22 SEN papers only) plus citations (for all papers) in the network.

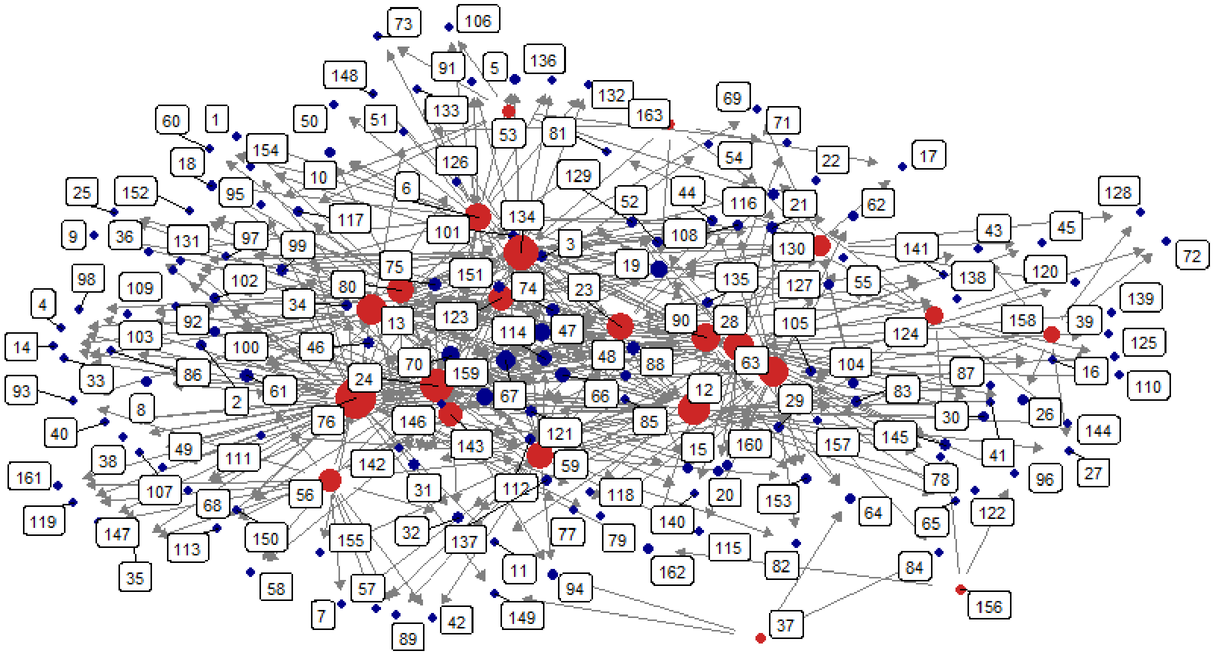

Fig S7. Reduced network diagram showing how the 22 SEN papers (red) are linked directly or through common citations (blue, n = 141; total network = 163 nodes). We removed all references that were only cited by a single SEN paper and thus, not uniting the network. Nodes are labeled using network ID values, which correspond to citations in table S1.

Table S1. Indegree centrality of papers among subnetwork of 163 papers with at least 2 citations. ID refers to the node ID in Fig S6

Indegree	Citation	ID
13	Folke_etal_2007.EcolSoc	67
11	Galaz_etal_2008.Chapter	70
11	Cumming_etal_2006.EcolSoc	47
11	Bodin_Tengo_2012.GEC	28
11	Bodin_etal_2014.ConservBiol	23
10	Young_2002.Book	159
9	Ekstrom_Young_2009.EcolSoc	59
9	Bodin_Crona_2009.GEC	19
8	Ostrom_1990.Book	114
8	Folke_etal_2005.AnnRevEnvRes	66
8	Bergsten_etal_2014.EcolSoc	12
7	Guerrero_etal_2013.ConservBiol	74
6	Treml_etal_2015.GEC	143
6	Janssen_etal_2006.EcolSoc	88
6	Guerrero_etal_2015.EcolSoc	76
6	Guerrero_etal_2015.ConservLett	75
6	Epstein_etal_2015.CurrOpinEnvSust	61
5	Wang_etal_2013.SocNet	151
5	Urban_Keitt_2001.Ecology	145
5	Pelosi_etal_2010.AgricEcosystEnviron	121
5	Ostrom_2009.Science	116
5	Lubell_etal_2014.EcolSoc	99
5	Kininmonth_etal_2015.AMBIO	90
5	Crowder_etal_2006.Science	46
5	Brown_2003.FrontEcolEnviron	32
5	Brondizio_etal_2009.AnnRevEnvRes	31
5	Bodin_etal_2006.EcolSoc	21
5	Armitage_etal_2009.FrontEcolEnviron	3
4	Wasserman_Faust_1994.Book	153
4	Sayles_Baggio_2017.PNAS	134
4	Robins_etal_2007.SocNet.Intro	129
4	Rathwell_Peterson_2012.EcolSoc	127
4	Olsson_etal_2007.EcolSoc	112
4	Milo_etal_2002.Science	105
4	MEA_2005.Report	104
4	Lusher_etal_2012.Book	100

4	Ernstson_etal_2010.EcolSoc	63
4	Cumming_etal_2010.DiversDistrib	48
4	Crona_Bodin_2006.EcolSoc	44
4	Cash_etal_2006.EcolSoc	34
3	Zetterberg_etal_2010.LandscapeUrbanPlann	162
3	Young_etal_2006.EcolSoc	160
3	Snijders_etal_2006.SociolMethodol	137
3	Schneider_etal_2003.AmJPolSci	135
3	Ostrom_2010.GEC	117
3	Newig_etal_2010.EcolSoc	108
3	McAllister_etal_2015.EcolSoc	102
3	McAllister_etal_2014.RegEnvChange	101
3	Levin_1998.Ecosystems	94
3	Hanneman_Riddle_2005.Book	83
3	Folke_2006.GEC	64
3	Ernstson_etal_2008.EcolSoc	62
3	Dietz_etal_2003.Science	52
3	Borgstrom_etal_2006.EcolSoc	30
3	Borgatti_etal_2009.Science	29
3	Bodin_Norberg_2007.LandscapeEcol	26
3	Bodin_etal_2016.EcolSoc	24
3	Bodin_etal_2006.EcolAppl	20
3	Bodin_2017.Science	18
3	Berkes_etal_2003.Book	15
3	Berardo_Scholz_2010.AmJPolitSci	10
3	Berardo_2014.PSJ	8
3	Baggio_etal_2016.PNAS	5
3	Ansell_Gash_2008.JPubAdminResTheory	2
2	Young_etal_2008.Book	161
2	Yletyinen_etal_2016.ProcRoyalSocBioSci	157
2	Wilson_2006.EcolSoc	155
2	Wheeler_2010.Rdocumentation	154
2	Wang_etal_2016.Chapter	152
2	Walker_etal_2009.Science	150
2	Walker_etal_2004.EcolSoc	149
2	Vignola_etal_2013.EnvironSciPolicy	148
2	Vatn_Vedeld_2012.EcolSoc	147
2	Vance-Borland_Holley_2011.ConservLett	146
2	Urban_etal_2009.EcologyLett	144

2	Termeer_etal_2010.EcolSoc	142
2	Tengo_von-Heland_2011.Chapter	141
2	Tengo_etal_2007.AMBIO	140
2	Stouffer_Bascompte_2011.PNAS	139
2	Stafford_etal_2009.Environment	138
2	Schoon_etal_2014.EnvironModelSoftw	136
2	Sayles_Baggio_2017.JEMA	133
2	Salau_etal_2012.EcolModell	132
2	Sabatier_etal_2005.Book	131
2	RCoreTeam_2013.Vienna	128
2	Prugh_etal_1999.Book	126
2	Prell_etal_2009.SocNatResour	125
2	Pickett_etal_2001.AnnuRevEcolEvolSyst	122
2	Pahl-Wostl_etal_2007.EcolSoc	120
2	Pahl-Wostl_2009.GEC	119
2	Ostrom_etal_1999.Science	118
2	Ostrom_2005.Book	115
2	Osterblom_Bodin_2012.ConservBiol	113
2	North_1990.Book	111
2	Newman_Dale_2005.EcolSoc	110
2	Newig_Fritsch_2009.EnvironPolGov	109
2	MunckafRosenschold_etal_2014.EcolSoc	107
2	Mucha_etal_2010.Science	106
2	McAllister_etal_2015.PSJ	103
2	Lubell_2013.PSJ	98
2	Lomi_etal_2016.SocNet	97
2	Lofvenhaft_etal_2004.LandscapeUrbanPlann	96
2	Liu_etal_2007.Science	95
2	Lebel_etal_2013.EcolSoc	93
2	Lazega_Snijders_2015.Book	92
2	Kivela_etal_2014.JComplexNetw	91
2	Juda_Hennessey_2001.OceanDevIntLaw	89
2	Ingo_etal_2006.Report	87
2	Hughes_etal_2013.TREE	86
2	Hooghe_Marks_2003.AmPolitSciRev	85
2	Holling_1996.Chapter	84
2	Hanna_etal_1996.Book	82
2	Handcock_etal_2008.JStatSoftw	81
2	Hahn_etal_2006.HumEcol	79

2	Gunnarsson_Lofroth_2009.Report	78
2	Gunderson_Holling_2002.Book	77
2	Granell_etal_2013.PhysRevLett	73
2	Girvan_Newman_2002.PNAS	72
2	Garmestani_Benson_2013.EcolSoc	71
2	Freeman_1979.SocNet	69
2	Frank_Strauss_1986.JAmStatAssoc	68
2	Folke_etal_1998.WorkingPaper	65
2	Epanchin-Niell_etal_2010.FrontEcolEnviron	60
2	Ekstrom_etal_2010.CoastManage	58
2	Ekstrom_etal_2009.MarPolicy	57
2	Duit_Galaz_2008.Governance	55
2	Duit_etal_2010.GEC	54
2	Dee_etal_2017.TREE	51
2	DeDomenico_etal_2016.NatPhys	50
2	Dallimer_Strange_2015.TREE	49
2	Crona_Bodin_2010.EcolSoc	45
2	Crona_2006.EcolSoc	43
2	Costanza_Folke_1996.Chapter	42
2	Costanza_etal_2006.Report	41
2	Cosens_2013.EcolSoc	40
2	Cinner_Bodin_2010.PlosOne	39
2	Christensen_etal_1996.EcolAppl	38
2	Chades_etal_2011.PNAS	36
2	Cash_Moser_2000.GEC	35
2	Carlsson_Berkes_2005.JEMA	33
2	Bodin_Prell_2011.Book	27
2	Bodin_Nohrstedt_2016.GEC	25
2	Bodin_etal_2011.Chapter	22
2	Biggs_etal_2012.AnnRevEnvRes	17
2	Berkes_Folke_1998.Book	16
2	Berkes_2009.JEMA	14
2	Bergsten_etal_2013.BiolConserv	11
2	Berardo_Lubell_2016.PublicAdmRev	9
2	Barnes_McFadden_2008.MarPolicy	7
2	Armitage_etal_2012.ConservLett	4
2	Ager_etal_2017.PlosOne	1
1	Roldán_etal_2015.EcosystServ	130
1	Pittman_Armitage_2017.EcolSoc	123

0	Zhao_etal_2018.CurrOpinEnvSust	163
0	Yletyinen_etal_2018.EcolSoc	158
0	Xiu_etal_2017.UrbanEcosyst	156
0	Prager_Pfeifer_2015.EcolSoc	124
0	Hamilton_etal_2019.GEC	80
0	Ekstrom_Crona_2017.SciTotalEnv	56
0	Dragicevic_Shogren_2017.AdvComplexSyst	53
0	Chopra_Khanna_2014.JEMA	37
0	Bergsten_etal_2019.EnvironSciPol	13
0	Baggio_Hillis_2018.EnvironModelSoftw	6

SI.5 Literature cited in the SI text (for brevity, omitting that from Table S1)

Barnes M L, Lynham J, Kalberg K and Leung P 2016 Social Networks and Environmental Outcomes *Proc. Natl. Acad. Sci. U.S.A.* **113** 6466–71

Berardo R and Scholz J T 2010 Self-Organizing Policy Networks: Risk, Partner Selection, and Cooperation in Estuaries *Am. J. Pol. Sci.* **54** 632–49 Online: http://doi.wiley.com/10.1111/j.1540-5907.2010.00451.x

Bergsten A, Jiren T S, Leventon J, Dorresteijn I, Schultner J and Fischer J 2019 Identifying governance gaps among interlinked sustainability challenges *Environ. Sci. Policy* **91** 27–38 Online: https://linkinghub.elsevier.com/retrieve/pii/S1462901118303010

Borgatti S P, Mehra A, Brass D J and Labianca G 2009 Network analysis in the social sciences. *Science (80-.).* **323** 892–5

Butts C T 2015 network: Classes for Relational Data_. The Statnet Project (<URL: http://statnet.org>). R package version 1.13.0.1, <URL: http://CRAN.R-project.org/package=network>.

Butts C T 2009 Revisiting the Foundations of Network Analysis *Science (80-.).* **325** 414–6

Butts C T 2016 sna: Tools for Social Network Analysis. R package version 2.4 https://CRAN.R-project.org/package=sna

Ekstrom J A and Young O R 2009 Evaluating functional fit between a set of institutions and an ecosystem *Ecol. Soc.* **14** 16

Groce J E, Farrelly M A, Jorgensen B S and Cook C N 2019 Using social-network research to improve outcomes in natural resource management *Conserv. Biol.* **33** 53–65

Hamilton M, Fischer A P and Ager A 2019 A social-ecological network approach for understanding wildfire risk governance *Glob. Environ. Chang.* **54** 113–23 Online: https://linkinghub.elsevier.com/retrieve/pii/S0959378017312232

Lubell M, Robins G and Wang P 2014 Network structure and institutional complexity in an ecology of water management games *Ecol. Soc.* **19** 23

Newig J, Günther D and Pahl-wostl C 2010 Synapses in the Network: Learning in Governance Networks in the Context of Environmental Management *Ecol. Soc.* **15** 24

Sayles J S and Baggio J A 2017a Social-ecological network analysis of scale mismatches in estuary watershed restoration *Proc. Natl. Acad. Sci. U.S.A.* **114**

Sayles J S and Baggio J A 2017b Who collaborates and why: Assessment and diagnostic of governance network integration for salmon restoration in Puget Sound , USA *J. Environ. Manage.* **186** 64–78 Online: http://dx.doi.org/10.1016/j.jenvman.2016.09.085

Turnbull L, Hütt M-T, Ioannides A A, Kininmonth S, Poeppl R, Tockner K, Bracken L J, Keesstra S, Liu L, Masselink R and Parsons A J 2018 Connectivity and complex systems: learning from a multi-disciplinary perspective *Appl. Netw. Sci.* **3** 11
